# A *Toxoplasma* Palmitoyl Acyl Transferase and the Palmitoylated Armadillo Repeat Protein TgARO Govern Apical Rhoptry Tethering and Reveal a Critical Role for the Rhoptries in Host Cell Invasion but Not Egress

**DOI:** 10.1371/journal.ppat.1003162

**Published:** 2013-02-07

**Authors:** Josh R. Beck, Connie Fung, Kurtis W. Straub, Isabelle Coppens, Ajay A. Vashisht, James A. Wohlschlegel, Peter J. Bradley

**Affiliations:** 1 Department of Microbiology, Immunology and Molecular Genetics, University of California, Los Angeles, Los Angeles, California, United States of America; 2 Department of Molecular Microbiology and Immunology, Johns Hopkins University Bloomberg School of Public Health, Baltimore, Maryland, United States of America; 3 Department of Biological Chemistry, David Geffen School of Medicine, University of California, Los Angeles, Los Angeles, California, United States of America; University of Vermont, United States of America

## Abstract

Apicomplexans are obligate intracellular parasites that actively penetrate their host cells to create an intracellular niche for replication. Commitment to invasion is thought to be mediated by the rhoptries, specialized apical secretory organelles that inject a protein complex into the host cell to form a tight-junction for parasite entry. Little is known about the molecular factors that govern rhoptry biogenesis, their subcellular organization at the apical end of the parasite and subsequent release of this organelle during invasion. We have identified a *Toxoplasma* palmitoyl acyltransferase, TgDHHC7, which localizes to the rhoptries. Strikingly, conditional knockdown of TgDHHC7 results in dispersed rhoptries that fail to organize at the apical end of the parasite and are instead scattered throughout the cell. While the morphology and content of these rhoptries appears normal, failure to tether at the apex results in a complete block in host cell invasion. In contrast, attachment and egress are unaffected in the knockdown, demonstrating that the rhoptries are not required for these processes. We show that rhoptry targeting of TgDHHC7 requires a short, highly conserved C-terminal region while a large, divergent N-terminal domain is dispensable for both targeting and function. Additionally, a point mutant lacking a key residue predicted to be critical for enzyme activity fails to rescue apical rhoptry tethering, strongly suggesting that tethering of the organelle is dependent upon TgDHHC7 palmitoylation activity. We tie the importance of this activity to the palmitoylated Armadillo Repeats-Only (TgARO) rhoptry protein by showing that conditional knockdown of TgARO recapitulates the dispersed rhoptry phenotype of TgDHHC7 knockdown. The unexpected finding that apicomplexans have exploited protein palmitoylation for apical organelle tethering yields new insight into the biogenesis and function of rhoptries and may provide new avenues for therapeutic intervention against *Toxoplasma* and related apicomplexan parasites.

## Author Summary

Apicomplexans possess a highly polarized secretory pathway that is critical for their ability to invade host cells and cause disease. This unique cellular organization enables delivery of protein cargo to specialized secretory organelles called micronemes and rhoptries that drive forward penetration into the host cell. The rhoptries are tethered in a bundle at the apex of the parasite, but how these organelles are organized in this manner is unknown. In this work, we identify a rhoptry-localized palmitoyl acyl transferase (named TgDHHC7) that functions to properly affix the rhoptries at the apical end of the parasite. Conditional disruption of TgDHHC7 results in a failure to tether the rhoptries at the cell apex and a corresponding loss of rhoptry function. We exploit this mutant to clearly demonstrate a critical role for the rhoptries in host invasion but not attachment or egress. Additionally, we find that mutation of a key residue predicted to be required for catalytic activity renders TgDHHC7 non-functional and that knockdown of the candidate substrate TgARO produces an identical phenotype to loss of TgDHHC7. The finding that *Toxoplasma* employs protein palmitoylation to position the rhoptries at the cell apex provides new insight into the molecular mechanisms that underlie apicomplexan cell polarity, host invasion and pathogenesis.

## Introduction

Apicomplexans are a large phylum of globally important parasites that cause substantial disease in their human and animal hosts. Species of particular interest to human health include *Toxoplasma gondii*, which infects one-third of the world's human population and causes disease in immunocompromised individuals and neonates, and *Plasmodium falciparum*, which causes malaria resulting in 1–2 million deaths annually [1], [2]. Most apicomplexans have obligate intracellular life cycles and the disease they cause is dependent upon their ability to actively invade their host cells, replicate within an intracellular niche and ultimately egress from the host. These key aspects of apicomplexan biology are critically linked to unique parasite-specific organelles and the remarkable polarized organization of the apicomplexan cell [3].

Host cell invasion is a highly coordinated process of attachment and penetration involving two apical secretory organelles, the micronemes and rhoptries [4]. The micronemes are small, bar-shaped organelles that line the apical third of the parasite periphery. To initiate the invasion process, an array of molecular adhesins are released from the micronemes onto the parasite surface and facilitate attachment to the host cell. On the cytosolic side of the parasite's plasma membrane, these adhesins are connected to an actin-myosin motor that is itself immobilized in a double-membrane system called the inner membrane complex (IMC). This motor apparatus (known as the glideosome) is responsible for generating a unique type of gliding motility that is utilized by apicomplexan parasites for host penetration [5].

Following attachment via micronemal adhesins, the parasites reorient to position their apical end towards the host membrane and the rhoptries are then released, an event that corresponds with the beginning of host penetration [4], [6]. Rhoptries are club-shaped organelles each consisting of a larger, bulbous body and tapered, duct-like neck [7]. The rhoptry necks are positioned at the extreme apex of the cell, providing a conduit for release of the organellar contents. At the onset of invasion, several proteins contained within the rhoptry necks are injected into the host membrane and localize to a tight-junction structure known as the “moving junction” through which the parasite passes to invade the host [8]. In contrast, proteins contained within the rhoptry body are injected into the host cytosol where they access various compartments and modulate host functions [7]. As the parasite penetrates the host, a parasitophorus vacuole is formed from invagination of the host membrane within which the parasite resides and replicates [9].

Protein traffic to the rhoptries and micronemes proceeds through a shared pathway until late in the secretory pathway [10]. General secretory pathway mutants have been described that eliminate the function of both the micronemes and rhoptries, illustrating the key importance of these organelles for motility, attachment, invasion and egress [10], [11]. While a mutant in the calcium-binding protein TgDOC2.1 has recently been described that fails to recruit the membrane fusion machinery needed for microneme exocytosis, mutants that specifically disable the rhoptries have not been reported [12]. Thus, the precise contribution of this organelle to invasion and egress remains to be clearly addressed. Additionally, while the highly polarized nature of the apicomplexan cell, including apical rhoptry positioning, has fascinated cell biologists for decades, the molecular mechanisms that establish this cell polarity are not understood.

Protein palmitoylation is a widely employed eukaryotic strategy for the spacio-temporal control of protein localization and function [13], [14]. Previous work in our lab and others showed that palmitoylated proteins play key roles in the organization and function of the IMC [15]–[17]. Additionally, the armadillo-repeat containing protein TgARO is recruited to the cytoplasmic face of the rhoptries via palmitoylation, indicating that this modification is also involved in rhoptry biology [18]. More broadly, a recently reported “palmitoylome” in *Plasmodium falciparum* revealed that this modification is extensively applied to parasite proteins implicated in many unique processes that are critical for pathogenesis [19]. Covalent attachment of palmitate to protein substrates is catalyzed by two palmitoyl acyl transferase (PAT) enzyme classes: the membrane bound O-acyl transferase (MBOAT) family and the Asp-His-His-Cys cysteine-rich domain (DHHC-CRD) family. Generally, MBOATs palmitoylate secreted proteins while DHHC-CRD PATs act on non-secreted substrates to recruit them to target membrane systems [20]. The *Toxoplasma* genome encodes three putative MBOAT family homologs. In contrast, 18 DHHC-CRD containing proteins are encoded, a relatively large number for a single-celled organism (mammals encode 23, *S. cerevisiae* encodes 7, *Giardia lamblia* encodes 9 and *Trypanosoma brucei* encodes 12), suggesting that palmitoylation of intracellular proteins has been extensively outfitted to serve the unique organelle systems in this parasite [21]–[23]. However, it should be noted that other apicomplexans appear to encode fewer DHHC-CRD proteins, such as *P. falciparum* which encodes 12. The localization and function of any of the DHHC-CRD proteins resident within apicomplexan-specific organelles remains to be determined.

In this work, we employ a candidate gene strategy to identify both rhoptry and IMC DHHC-CRD PATs in the model apicomplexan *Toxoplasma*. To explore the role of palmitoylation in rhoptry biology, we perform a conditional knockdown of the rhoptry PAT (named TgDHHC7). Interestingly, elimination of this enzyme results in a loss of apical rhoptry tethering with the organelles instead scattered throughout the cytosol. While the positioning of the rhoptries in this mutant is completely disrupted, the biogenesis, morphology and cargo sorting of the organelles are not impacted. Likewise, other secretory organelles are unaffected by loss of TgDHHC7. Parasites with dispersed rhoptries are completely unable to invade host cells, but motility, attachment and egress are not impacted, revealing the precise role of the rhoptries and demonstrating that their positioning is critical for function. We use mutagenesis to show that the divergent N-terminal region of TgDHHC7 is dispensable for both targeting and function while a short C-terminal region is required for rhoptry localization. A mutant version of TgDHHC7 lacking a key residue predicted to be required for catalytic activity fails to rescue rhoptry function, strongly suggesting that palmitoylation activity is required for tethering. Finally, we provide evidence that TgARO is a key substrate recruited by TgDHHC7 to facilitate rhoptry apical organization by showing that conditional knockdown of TgARO produces the same dispersed rhoptry phenotype that results from knockdown of TgDHHC7. These results demonstrate the importance of protein palmitoylation for apicomplexan biology and provide new insight into the polarization of the *Toxoplasma* cell and rhoptry biosynthesis and function.

## Results and Discussion

### Identification of palmitoyl acyl transferases targeted to the *Toxoplasma* rhoptries or IMC

**Figure 1:**
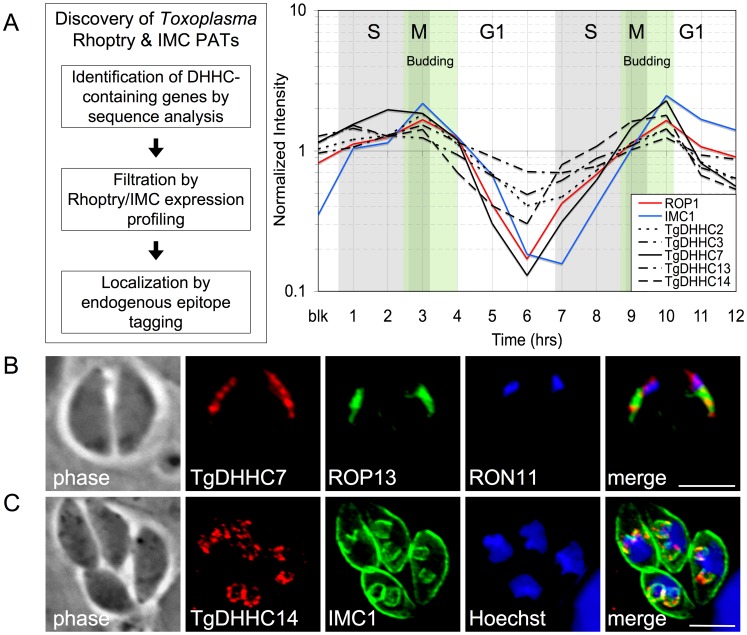
Identification of palmitoyl acyl transferases targeted to the *Toxoplasma* rhoptries or IMC. (A) The expression profile of 18 candidate PATs (DHHC-CRD containing genes) was compared to known IMC and rhoptry genes across the *Toxoplasma* cell cycle (Figure S1). Five candidate genes were found to display an expression signature similar to the IMC/rhoptry pattern, peaking in a narrow window during daughter cell budding. (B–C) IFA of two candidate IMC/rhoptry PATs localized by introduction of a 3xHA epitope tag at the endogenous C-terminus. (B) TgDHHC7 was found to localize to the rhoptries, as assessed by colocalization with both the rhoptry body protein ROP13 and rhoptry neck protein RON11. Red: anti-HA antibody detected by Alexa594-anti-mouse IgG. Green: rabbit anti-ROP13 antibody detected by Alexa488-anti-rabbit IgG Blue: rat anti-RON11 antibody detected by Alexa350-anti-rat IgG. (C) TgDHHC14 was found to localize to the IMC, as assessed by colocalization with the IMC protein IMC1. Red: anti-HA antibody detected by Alexa594-anti-rabbit IgG. Green: mouse anti-IMC1 antibody detected by Alexa488-anti-mouse IgG. Blue: Hoechst stain. All scale bars = 5 µm.

To explore the function of palmitoylation in parasite-specific processes, we sought to identify PATs resident in organelles unique to apicomplexans using the model apicomplexan *Toxoplasma*. Because we are primarily interesting in the recruitment of proteins to the cytoplasmic face of organelles [15], [18], we focused on the 18 DHHC-CRD homologs encoded within the *Toxoplasma* genome (Table S1). The rhoptries and IMC are assembled *de novo* during each round of parasite internal budding with expression of IMC and rhoptry genes peaking together in a narrow window, as shown by the expression profile of prototypical rhoptry and IMC genes (ROP1 and IMC1, Figure 1A). Thus, we filtered our candidate gene list by comparing expression-timing data for the 18 putative PATs with these representative genes (Figure 1A and Figure S1A). Of the highly transcribed candidate PATs, five genes displayed a rhoptry/IMC expression signature. We attempted to localize the corresponding proteins by introduction of a 3xHA epitope tag at the endogenous C-terminus of each gene and succeeded for two of the five candidate PATs. One of these proteins was found to localize to the rhoptries (TGME49_252200, named TgDHHC7) while the other localized to the IMC (TGME49_293730, named TgDHHC14), validating our approach (Figure 1B–C). In this work, we focus on functional characterization of the rhoptry-localized TgDHHC7 to gain insight into the contribution of palmitoylation activity to rhoptry biology.

A comparison of TgDHHC7 distribution with the rhoptry body protein ROP13 and rhoptry neck protein RON11 (TGME49_230350, Figure S2) shows colocalization with both markers, indicating that TgDHHC7 is present along the length of the entire rhoptry in both the bulbous body and duct-like neck (Figure 1B). To confirm the coding sequence for this gene, we cloned and sequenced the TgDHHC7 cDNA. While the resulting sequence agrees with RNAseq data [24], the migration of the endogenously tagged protein by SDS-PAGE is inconsistent with the predicted size, leaving ambiguity about the correct start codon, which we resolved with a combination of mass spectrometry and ectopic protein expression in *Toxoplasma* (Figure S3).

**Figure 2:**
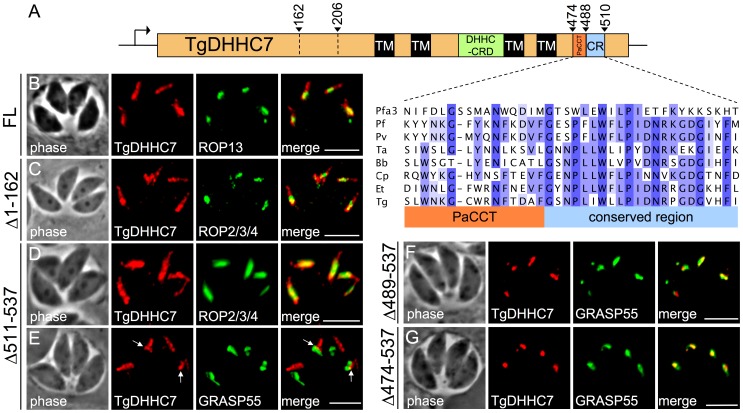
A conserved region of the TgDHHC7 C-terminus is required for rhoptry targeting. Analysis of determinants for rhoptry targeting of TgDHHC7. (A) Diagram showing different truncation mutants utilized in this study with an expanded view of the C-terminal PaCCT motif (red box) and downstream conserved region (CR, blue box). Homology between TgDHHC7 residues 474–510 and various orthologs is shown (see Figure S3 for full alignment details). (B) A second copy of the full length TgDHHC7 coding sequence with a C-terminal HA epitope tag was expressed in wild type parasites under the control of the RON5 promoter and found to localize to the rhoptries in the same fashion as the endogenous protein. The localization of several truncations mutants was then evaluated. Red: anti-HA antibody detected by Alexa594-anti-mouse IgG. Green: rabbit anti-ROP13 antibody detected by Alexa488-anti-rabbit IgG. (C) Removal of residues 1–162 had no apparent effect on rhoptry localization (Δ1–162). Red: anti-HA antibody detected by Alexa594-anti-rabbit IgG. Green: mouse anti-ROP2/3/4 antibody detected by Alexa488-anti-mouse IgG. (D) Truncation of residues 511–537 to remove a non-conserved extreme C-terminal region of the protein did not grossly impact targeting of TgDHHC7. Red: anti-HA antibody detected by Alexa594-anti-rabbit IgG. Green: mouse anti-ROP2/3/4 antibody detected by Alexa488-anti-mouse IgG. (E) However, in some parasites TgDHHC7_Δ511–537_ signal was detected outside the rhoptries adjacent to the Golgi marker GRASP55. Red: anti-HA antibody detected by Alexa594-anti-mouse IgG. Green: GRASP55-YFP. (F–G) In contrast, further C-terminal truncations that remove the CR (residues 489–537) or the CR and PaCCT motif (residues 474–537) completely abrogate rhoptry targeting. These mutants were found to localize to the Golgi, as assessed by co-localization with the GRASP55, indicating the CR is necessary for TgDHHC7 transit from the Golgi to its final rhoptry destination. Red: anti-HA antibody detected by Alexa594-anti-mouse IgG. Green: GRASP55-YFP. Scale bars = 5 µm.

TgDHHC7 is predicted to contain four transmembrane helices with the highly conserved DHHC residues located just upstream of the third of these hydrophobic regions (Figure 2A). DHHC-CRD PATs characterized in other systems function to palmitoylate soluble, cytosolic proteins, tethering them to target membranes. Thus, TgDHHC7 likely adopts a membrane topology that positions the DHHC-CRD domain in the cytosol where it would enable anchoring of proteins to the cytosolic face of the rhoptries. In agreement with this expected topology, TgARO was recently shown to traffic to the cytosolic face of the rhoptry membrane via protein palmitoylation [18].

Orthologs to TgDHHC7 were identified across the apicomplexan phylum, indicating conservation in related organisms possessing rhoptries (Figure S4). Conservation beyond Apicomplexa was also observed, including homology to the yeast PAT Pfa3, which is involved in the recruitment of Vac8p to the yeast vacuole to facilitate homotypic membrane fusion [25], [26]. Interestingly, Vac8p also contains armadillo repeats and is homologous to TgARO [18], further suggesting that TgARO may be recruited to the rhoptries by TgDHHC7 and raising the possibility of a role in rhoptry membrane fusion for this putative enzyme-substrate pair. Additionally, the acidic environment of the rhoptry lumen has led to the proposal that rhoptries may be a form of secretory lysosomal granules [27]. The fact that both TgDHHC7 and TgARO are homologous to the Pfa3/Vac8p proteins of the yeast vacuole, the equivalent of a lysosome in this organism, further suggests that rhoptries in apicomplexan parasites resemble specialized lysosomes.

### A conserved C-terminal region is required for rhoptry trafficking of TgDHHC7

Although the targeting determinants for a number of soluble rhoptry proteins have been characterized, less is known about the requirements for trafficking of multipass transmembrane proteins to the rhoptries. While the core DHHC-CRD and surrounding TM domains are present in all known PATs, the N- and C-terminal regions outside of the TM domains are highly variable and thought to play roles in subcellular targeting and substrate recognition specific to individual PATs [28]. TgDHHC7 has a long N-terminal region upstream of the first TM domain (residues 1–247) and a shorter C-terminal region downstream of the fourth TM domain (residues 456–537) (Figure 2A). The N-terminal region of TgDHHC7 is elongated and divergent relative to orthologs in other apicomplexans (Figure S4). In contrast, the TgDHHC7 C-terminal region appears well-conserved among these orthologs, as well as with yeast Pfa3 (Figure S4). The C-terminal region of TgDHHC7 includes a PaCCT motif (red box, Figure 2A), a recently identified motif present in most eukaryotic PATs that is important for targeting in Pfa3 [29]. Downstream of the PaCCT motif is a conserved stretch of 21 residues (blue box, Figure 2A) followed by a less conserved region at the extreme C-terminus.

To test the importance of these features in rhoptry targeting of TgDHHC7, we expressed a second copy of TgDHHC7 cDNA with a C-terminal HA epitope tag under the control of the promoter for the rhoptry neck protein RON5, which has a similar expression profile to the endogenous TgDHHC7 promoter. This full-length version of TgDHHC7 targets properly to the rhoptries (Figure 2B). We then generated a series of N- and C-terminal truncations in this second copy of TgDHHC7 and evaluated their impact on targeting. A TgDHHC7 truncation mutant lacking the majority of the N-terminal region (residues 1–162) had no defect in rhoptry localization (Figure 2C). A more extensive N-terminal truncation (residues 1–206) also targeted properly (data not shown), demonstrating that the N-terminus of TgDHHC7 is not required for targeting.

We next evaluated the importance of the various C-terminal features of TgDHHC7 for rhoptry trafficking. Removal of the non-conserved, extreme C-terminal region (residues 511–537, Figure S4) did not grossly affect targeting (Figure 2D), although this mutant was sometimes also seen in a non-rhoptry compartment that localized adjacent to the Golgi marker GRASP55 (arrows, Figure 2E). In contrast, truncation of the highly conserved region up to but not including the PaCCT motif (residues 489–537) or including the PaCCT motif (residues 474–537) resulted in a complete loss of rhoptry localization, showing these residues are necessary for trafficking TgDHHC7 to the rhoptries (Figure 2F–G). These truncated proteins were found to colocalize with GRASP55, indicating TgDHHC7 fails to traffic beyond the Golgi in mutants lacking these key residues. Together, these results show that the TgDHHC7 C-terminal conserved region (blue, Figure 2A) just downstream of the PaCCT motif plays a key role in rhoptry sorting.

### Establishment of a TgDHHC7 conditional knockdown mutant

**Figure 3:**
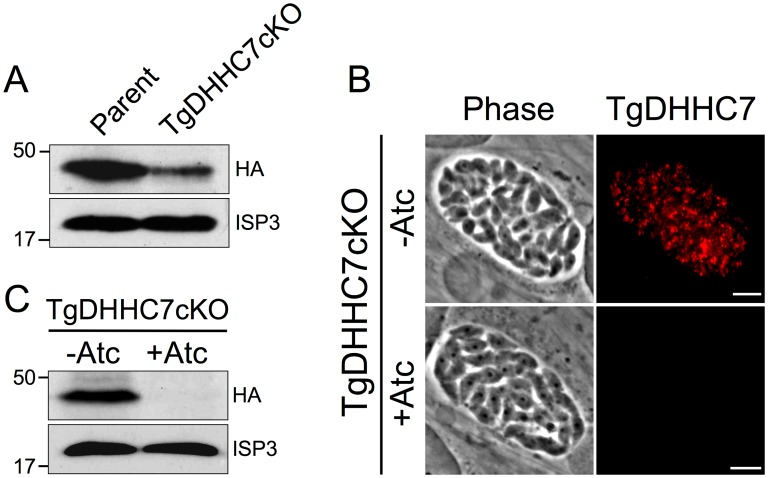
Establishment of a TgDHHC7 conditional knockdown. (A) Western blot comparing TgDHHC7 protein levels in parental and TgDHHC7cKO strains without Atc treatment. Exchange of the endogenous TgDHHC7 promoter with a Tet-repressible, conditional promoter results in lower levels of basal TgDHHC7 expression. ISP3 serves as a loading control. (B–C) Growth of TgDHHC7cKO parasites in the presence of Atc results a depletion of TgDHHC7. (B) Western blot showing TgDHHC7 levels after 72 hours −/+ Atc. (C) IFA showing TgDHHC7 signal after 48 hours −/+ Atc. Red: anti-HA antibody detected by Alexa594-anti-mouse IgG. Scale bars = 5 µm.

Localization of TgDHHC7 to the rhoptries indicates that *Toxoplasma* utilizes protein palmitoylation for specialized parasite secretory functions. To directly assess the role of TgDHHC7 in rhoptry biology, we replaced the endogenous promoter with a tetracycline-repressible promoter in TgDHHC7-3xHA parasites via homologous recombination to generate a conditional knockdown mutant, which we named TgDHHC7cKO. As expected, exchange of the endogenous promoter with a weaker truncated version of the SAG4 promoter results in lower TgDHHC7 protein levels than those observed in the parental line (Figure 3A). Rhoptries are assembled *de novo* during each round of parasite division and protein traffic to the organelle is restricted to a narrow window during biosynthesis. In agreement with this, we observe some mistargeting of TgDHHC7 under the control of the constitutive SAG4 promoter in the TgDHHC7cKO parasites, which likely corresponds to protein synthesized outside of this rhoptry biosynthesis timeframe (−Atc, Figure 3B). Culture of TgDHHC7cKO parasites in the presence of anhydrotetracycline (Atc) results in depletion of TgDHHC7 with protein levels falling below detectable levels by 72 hours (Figure 3B–C).

### TgDHHC7 is required for apical docking during rhoptry biogenesis

**Figure 4:**
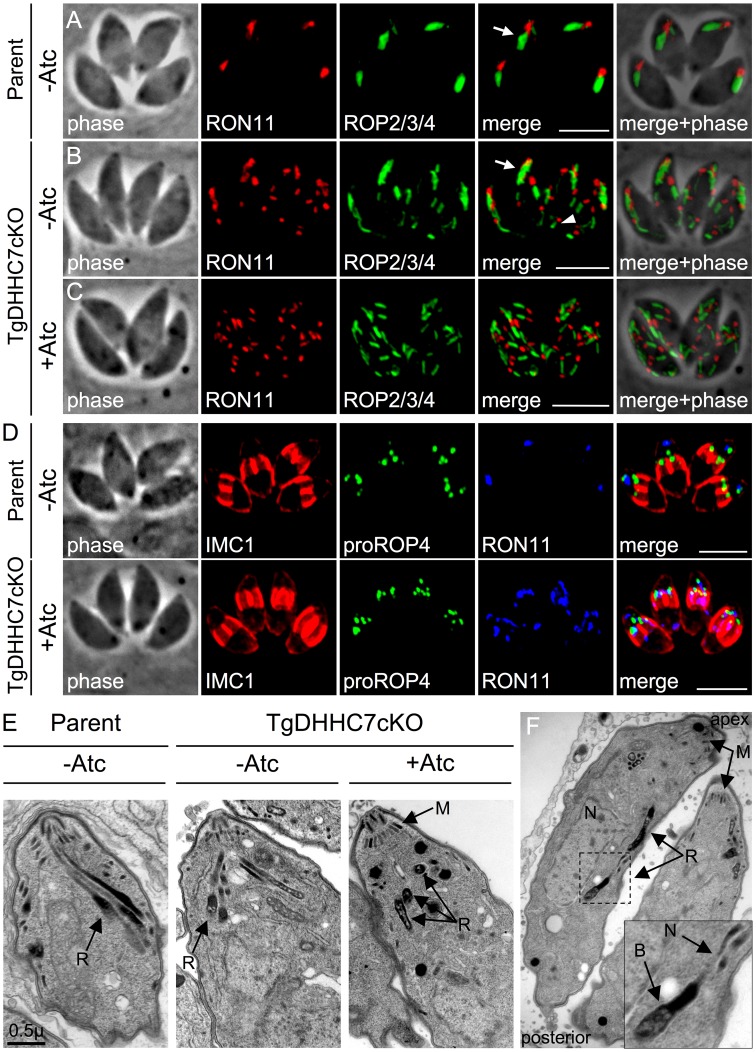
TgDHHC7 is required for apical rhoptry docking. (A–C) IFA detecting the rhoptry body protein ROP2/3/4 and rhoptry neck protein RON11. (A) During normal rhoptry biogenesis, 6–14 rhoptries are generated, each with a polarized morphology consisting of a posterior, bulbous body and tapered anterior neck domain. These individual rhoptries are bundled together with the necks docked at the extreme apical end of the parasite (arrow). (B) TgDHHC7cKO parasites, which express lower levels of TgDHHC7 than the parental line, maintain an apical bundle of rhoptries (arrow) but also contain some individual rhoptries in posterior areas of the cell (arrowhead). (C) Upon depletion of TgDHHC7 by Atc treatment, apical rhoptry bundles are lost with individual rhoptries scattered throughout the cell cytosol. The rhoptry neck and body domains are clearly maintained in each scattered organelle. Red: rat anti-RON11 antibody detected by Alexa594-anti-rat IgG. Green: mouse anti-ROP2/3/4 antibody detected by Alexa488-anti-mouse IgG. All IFA scale bars = 5 µm. (D) Rhoptry organization defects incurred by loss of TgDHHC7 occur after pro-rhoptry formation. Mature rhoptries labeled by RON11 are scattered throughout the cell following Atc treatment of TgDHHC7cKO parasites. In contrast, the pro-rhoptry marker proROP4 is only observed within forming daughter buds labeled by IMC1, similar to what is observed in the parental line. Red: anti-IMC1 antibody detected by Alexa594-anti-mouse IgG. Green: rabbit anti-proROP4 antibody detected by Alexa488-anti-rabbit IgG Blue: rat anti-RON11 antibody detected by Alexa350-anti-rat IgG. (E–F) TEM analysis of parent and TgDHHC7cKO lines. (E) A normal apical bundle of rhoptries (R) are visible in wild-type parasites and in untreated TgDHHC7cKO cells. Following Atc treatment of TgDHHC7cKO parasites, bundles of rhoptries docked at the extreme apex of the cell are no longer observed. Rhoptries are instead scattered, with many no longer oriented in a longitudinal fashion (R). Importantly, a standard apical arrangement of micronemes (M) is still present in these conditions. (F) In TgDHHC7 depleted cells, individual rhoptries are observed scattered throughout the cell cytosol. These scattered rhoptries are morphologically unchanged with obvious body (B) and neck (N) domains. Note the lumen of these rhoptries have a normal, mottled appearance.

*Toxoplasma* parasites possess 6–14 individual rhoptries each composed of a larger posterior body and tapered anterior neck [30]. To determine the effect of the loss of TgDHHC7 on the rhoptries, we performed IFA analysis on TgDHHC7cKO with an array of antibodies recognizing components of either the rhoptry body (ROPs) or rhoptry neck (RONs). As seen in the parental line, the rhoptries are normally bundled together in the apical end of the cell with the necks of the organelle docked at the extreme apex of the parasite where they are positioned to facilitate exocytosis of their contents (Figure 4A, arrow) [30], [31]. TgDHHC7cKO parasites cultured without Atc maintained the apical rhoptry bundle (Figure 4B, arrow) but also contained some individual rhoptries present outside of the apical region of the cell (Figure 4B, arrowhead), presumably due to the lower levels of TgDHHC7 produced in this line. In contrast, following culture with Atc, TgDHHC7cKO parasites showed a complete loss of the apical rhoptry bundle resulting in dispersed rhoptries scattered throughout the cell (Figure 4C and Movie S1). Interestingly, these scattered rhoptries appear to retain normal morphology and subdomain organization with each ROP2/3/4 body signal associated with a single, polar RON11 neck signal. Standard morphology and subdomain organization was also observed in these dispersed rhoptries with an array of additional rhoptry markers including the body markers ROP7, ROP13 and PP2Chn and the neck markers RON2 and RON8 (data not shown). In contrast, no effect was observed with IFA markers for the micronemes (Figure S6A), IMC (Figure 4D), dense granules, mitochondrion, apicoplast or plant-like vacuole (data not shown), indicating these organelles are unaffected by loss of TgDHHC7. The protein contents of scattered rhoptries also appeared normal in regard to maturation by proteolytic processing of both ROPs and RONs (Figure S5A).

Rhoptries are synthesized during each round of *Toxoplasma* replication wherein new daughter cells are assembled inside an intact mother cell [32]. To determine the point at which parasites depleted of TgDHHC7 incur rhoptry defects, we examined relevant markers for rhoptry biogenesis by IFA. Late secretory compartments labeled by the dynamin-like protein DrpB that are specifically destined for rhoptry/microneme organelles appeared normal (Figure S5B), as did markers for pro-rhoptries (Figure 4D), indicating that traffic to the dispersed rhoptries proceeds normally [11], [33]. During parasite replication, the nascent rhoptries are detected in early forming daughter parasites, suggesting that tethering may be critical for organellar delivery into daughter cells. However, while mature rhoptries in parasites lacking TgDHHC7 are not docked at these structures, biogenesis of new rhoptries faithfully occurs within assembling daughter buds and not elsewhere in the maternal cell (Figure 4D). This observation indicates that the position of rhoptry nucleation is likely a consequence of golgi/ER polarization at the base of daughter buds and that tethering at the apex of daughter buds is a later event in rhoptry biogenesis. Taken together, these results suggest that proteins recruited to the rhoptry surface by TgDHHC7 play critical roles in proper rhoptry bundling and apical docking, but not organelle assembly or sorting of rhoptry contents through the secretory pathway.

To better assess the morphology and orientation of rhoptries in the TgDHHC7 knockdown, we analyzed these parasites by transmission electron microscopy (TEM). Apical bundles of rhoptries properly docked within the conoid were easily observed in both the parental line and untreated TgDHHC7cKO parasites while apical rhoptry localization and docking were almost never observed following Atc treatment (Figure 4E). Under these conditions, scattered rhoptries were observed in the knockdown throughout the cytosol distal to the parasite apex (Figure 4F). In agreement with our IFA analysis, these scattered rhoptries appeared morphologically normal with large, mottled bodies and tapered necks (inset, Figure 4F). Other aspects of the cell ultrastructure appeared normal, including the presence and proper positioning of micronemes (Figure 4E–F). Less frequently, some irregular structures could be observed in parasites depleted of TgDHHC7, including the presence of amylopectin granules (observed in <9% of sections) and multi-membranous structures of unclear origin (observed in <7% of sections) (Figure S5C–D). Overall, these results demonstrate that loss of TgDHHC7 causes a severe and specific defect in apical rhoptry docking.

### Rhoptry function is critical for host cell invasion but not egress

**Figure 5:**
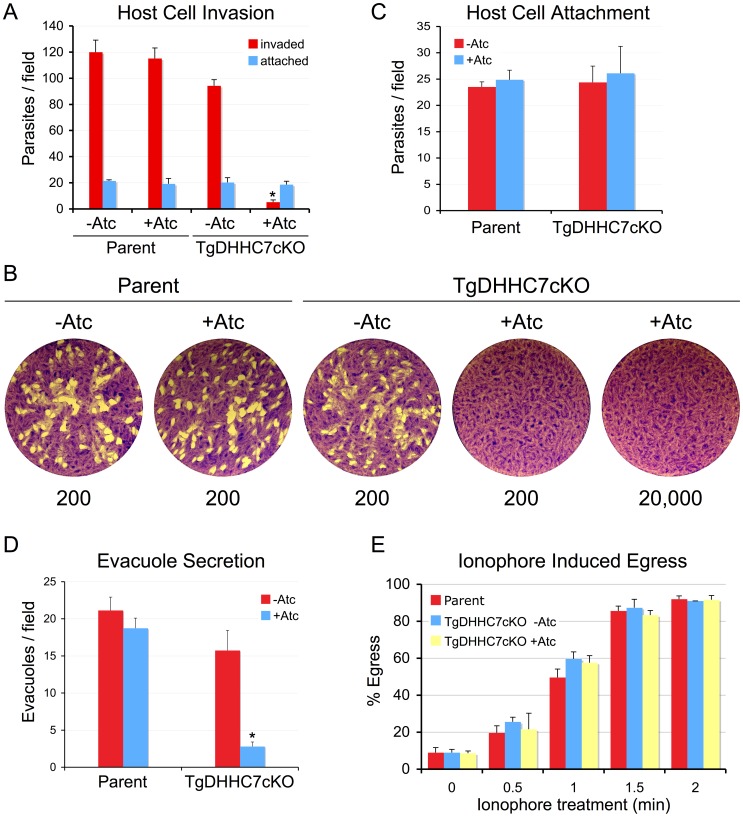
TgDHHC7 is critical for host invasion but not egress. (A–B) Parasites depleted of TgDHHC7 encounter a complete block in invasion. (A) Parental or TgDHHC7cKO parasites were grown for 72 hours−/+Atc and then allowed to invade into fresh host cells for one hour. Following depletion of TgDHHC7, parasites show a nearly complete block in host penetration (asterisk, p-value<0.001). A corresponding increase in attached, uninvaded parasites is not seen (blue bars). A minor decrease in penetration is also seen for untreated TgDHHC7cKO, likely due to the lower levels of TgDHHC7 expressed in this strain relative to the parental line. (B) Parasites depleted of TgDHHC7 cannot form plaques in fibroblast monolayers. Parental or TgDHHC7cKO parasites were grown 48 hours−/+Atc and then infected into fresh fibroblast monolayers at an infective dose of 200 parasites per well and allowed to incubate for nine days. TgDHHC7cKO parasites are unable to form plaques in the presence of Atc, even at an infective dose of 20,000 parasites per well. (C) Initial attachment is not affected upon knockdown of TgDHHC7. Parasites were grown for 60 hours−/+Atc before treatment with cytochalasin D to block motility and arrest the invasion process just after attachment. (D) Loss of TgDHHC7 impairs secretion of evacuoles by rhoptries (asterisk, p-value<0.001). Parasites were grown for 60 hours−/+Atc before treatment with cytochalasin D to block invasion and allow evacuole formation. Evacuoles were detected by staining for ROP2/3/4. (E) Parasite egress is unaffected by knockdown of TgDHHC7. Parasites were grown 60 hours−/+Atc and then induced to egress by treatment with calcium ionophore A23187 before fixation and staining for detection with anti-SAG1. The egress efficiency of parasites with defective rhoptries (lacking TgDHHC7) was not significantly different from parental or untreated TgDHHC7cKO parasites.

Host cell invasion by apicomplexan parasites involves the establishment of a tight-junction interface called the moving junction between the host and parasite surface during penetration [34]. Formation of the moving junction is believed to depend on a complex of rhoptry neck proteins injected into the host cell just before penetration [8]. While rhoptries in the TgDHHC7 knockdown appear morphologically intact and receive their proper contents, their failure to dock at the cell apex suggests these organelles are unable to secrete their contents and are thus rendered non-functional. Indeed, while parasites depleted of TgDHHC7 grow normally within the host cell (data not shown), they encounter a nearly complete block in invasion of new host cells (Figure 5A). The few invasion events observed following TgDHHC7 knockdown are likely the result of residual levels of TgDHHC7 as when the knockdown is extended over several lytic cycles, TgDHHC7cKO parasites are unable to form plaques, demonstrating that proper rhoptry secretion is critical for invasion and thus for survival of the parasite (Figure 5B).

Interestingly, the invasion block encountered upon knockdown of TgDHHC7 is not accompanied by a corresponding increase in parasites attached to the host surface (Figure 5A, blue bars). To ensure that secretion of micronemal adhesins is not compromised, we further evaluated the integrity and function of this organelle by IFA and gliding motility assays and found no detectable defects compared to wild-type parasites (Figure S6). A similar phenotype is seen upon disruption of the moving junction component RON8 in which initial attachment is unaffected, but parasites that fail to invade subsequently detach and are washed away in the assay [35]. To determine if this is the case in the TgDHHC7cKO strain, we treated parasites with cytochalasin D to paralyze the actin-myosin motor that powers invasion in order to separate initial attachment from subsequent invasion events. Under these conditions, no difference in attachment was seen for cells depleted of TgDHHC7, indicating that initial attachment is not impacted and that parasites lacking functional rhoptries detach from the host following failure to invade (Figure 5C).

At the onset of invasion, *Toxoplasma* parasites inject a subset of rhoptry body proteins into their host cell in structures called evacuoles, which can be visualized by IFA [36]. To ensure injection was blocked from rhoptries that fail to dock at the cell apex, we evaluated evacuole formation and found these structures were largely abolished in the absence of TgDHHC7 (reduced ∼82% in TgDHHC7cKO following Atc treatment), confirming a defect in exocytosis of rhoptry proteins (Figure 5D). Collectively, these results indicate that rhoptry secretion and host penetration are dependent on TgDHHC7-mediated apical docking. Additionally, the specific ablation of rhoptry function without impacting the other regulated secretory organelles provides the first unequivocal demonstration that rhoptry apical secretion is critical for invasion.

In addition to their role in invasion, rhoptries have also been suggested to function in egress as RON4-postive moving junction rings have been observed during host exit [37]–[39]. On the other hand, pharmacological inhibition of phospholipase activity blocks rhoptry secretion and inhibits invasion but not egress; however, these results are difficult to interpret in light of clear pleiotropic effects and a lack of any identified target(s) [40]. To resolve the question of rhoptry contribution to egress, we evaluated the ability of TgDHHC7-depleted parasites to egress and observed no obvious defects in natural egress (data not shown). To more quantitatively assess this question, we induced egress using the calcium ionophore A23187 and again observed no significant difference in egress efficiency between parasites with or without functional rhoptries (Figure 5E), clearly demonstrating that rhoptries are not important for host cell exit.

### TgDHHC7 palmitoyl transferase activity is required for apical rhoptry docking

**Figure 6:**
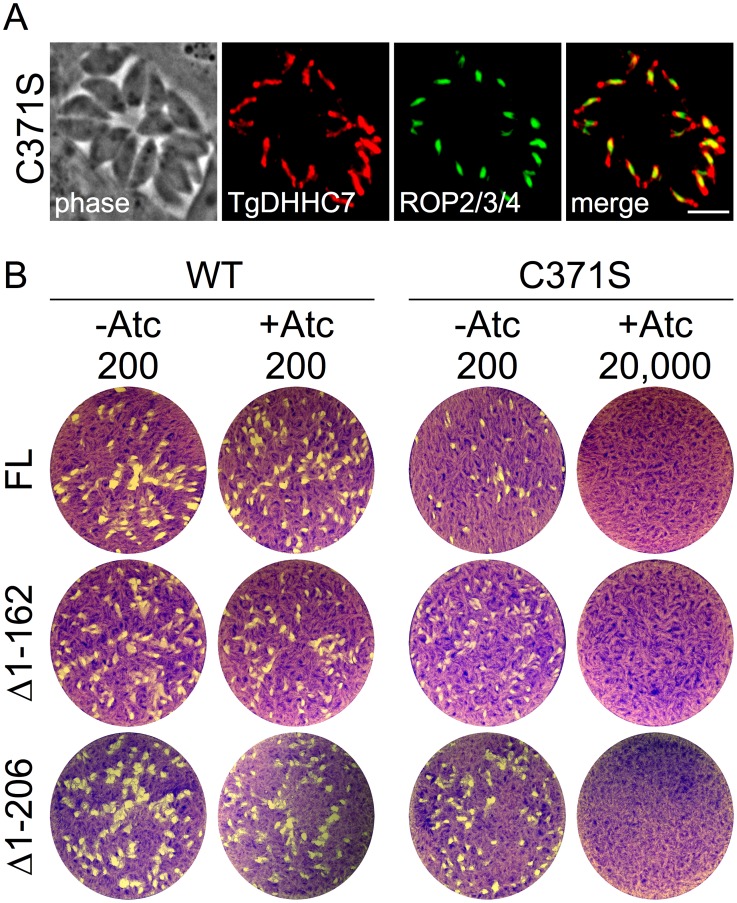
A key cysteine residue predicted to be required for TgDHHC7 catalytic activity is necessary for rhoptry function. (A) IFA showing localization of C371S mutant version of TgDHHC7. Targeting to the rhoptries is unaffected by the C371S mutation as assessed by co-localization with the rhoptry body protein ROP2/3/4. Red: anti-HA antibody detected by Alexa594-anti-rabbit IgG. Green: mouse anti-ROP2/3/4 antibody detected by Alexa488-anti-mouse. Scale bar = 5 µm. (B) Complementation of TgDHHC7cKO assessed by plaque assay. TgDHHC7_FL-WT_ rescues the defect incurred by the knockdown of TgDHHC7 while TgDHHC7_FL-C371S_ does not, failing to form plaques even with an infectious dose of 20,000 parasites per well. TgDHHC7 N-terminal truncations removing the first 162 or 206 residues also rescue the defect while C371S mutant versions of these truncation mutants do not.

Palmitoylation by membrane-resident PATs is a well-characterized mechanism for recruiting proteins to a target membrane system [13], [14]. Therefore, it is likely that TgDHHC7 facilitates apical docking of rhoptries by recruiting one or more proteins to the cytosolic face of the rhoptry membrane which then serve to mediate docking. Alternatively, it is possible that TgDHHC7 directly mediates docking in a manner that is independent of its palmitoylation activity. To distinguish between these possibilities, we generated a mutant version of TgDHHC7 by changing the cysteine within the highly conserved DHHC to a serine (TgDHHC7_C371S_). This mutation has been shown to abolish palmitoylation activity *in vitro* and function *in vivo* for several characterized PATs, including Pfa3 [25], [41], [42]. We then complemented our TgDHHC7cKO mutant by targeting a second copy of either the wild-type or mutant TgDHHC7 expression cassette to the *Toxoplasma* uracil phosphoribosyl transferase locus. While both versions of TgDHHC7 localize to the rhoptries (Figure 6A), only complementation with wild-type is able to rescue the lethal block in invasion incurred upon knockdown of the endogenous copy of the gene (Figure 6B, FL). These results strongly suggest that TgDHHC7 catalytic activity is necessary for rhoptry tethering through the recruitment or modification of other factors.

Since truncation of the first 162 residues of TgDHHC7 does not affect rhoptry targeting (Figure 2C), we complemented the TgDHHC7cKO strain with TgDHHC7_Δ1–162_ and found it is able to functionally complement the loss of endogenous TgDHHC7, as shown by the formation of plaques in the presence of Atc (Figure 6B, Δ1–162). A further truncation of TgDHHC7 lacking the first 206 resides (TgDHHC7_Δ1–206_) also targets properly to the rhoptries and rescues invasion upon knockdown of the endogenous TgDHHC7 (Figure 6B, Δ1–206). As expected, C371S mutant versions of TgDHHC7_Δ1–162_ and TgDHHC7_Δ1–206_ target to the rhoptries (not shown) but fail to rescue the knockdown defect. These results demonstrate that the N-terminal region of TgDHHC7 (residues 1–206) is not necessary for proper rhoptry targeting or function.

### TgARO is required for apical rhoptry tethering

**Figure 7:**
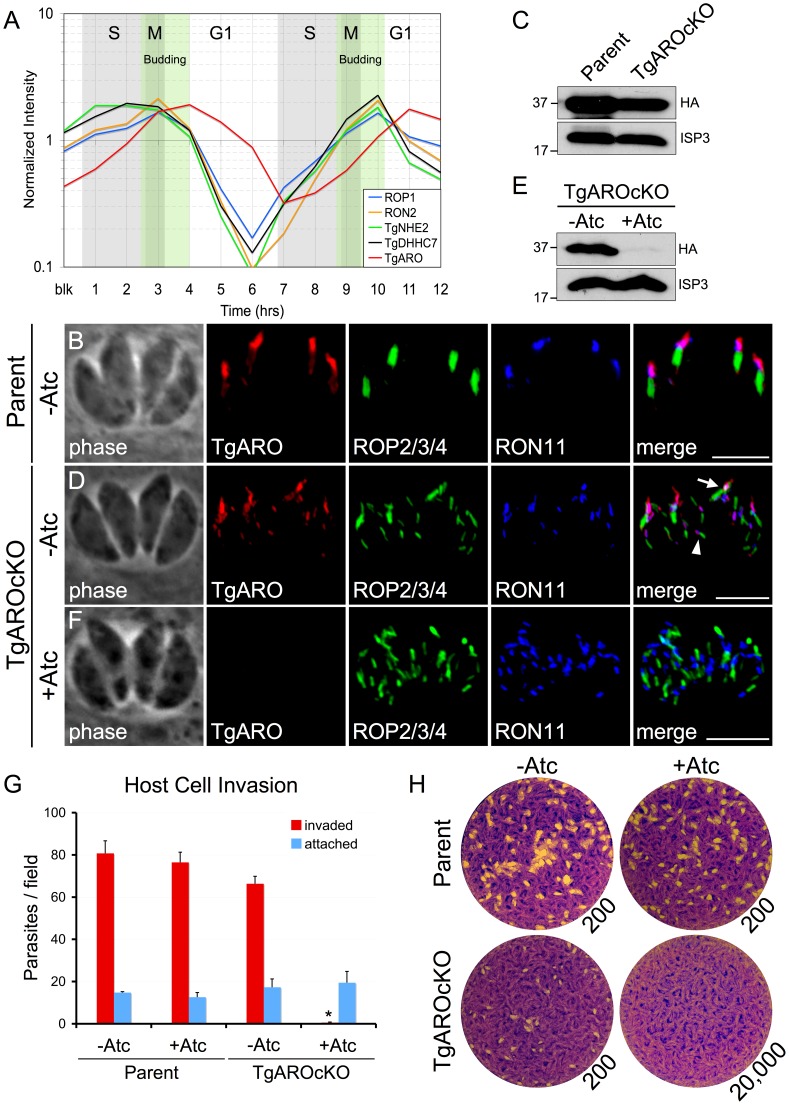
TgARO is required for apical rhoptry tethering and host cell invasion. (A) Expression of TgARO is delayed about one hour after other rhoptry proteins. Expression of representative genes from several classes of rhoptry proteins are shown including ROP1 (rhoptry body proteins), RON2 (rhoptry neck proteins) and the Na+/H+ exchanger TgNHE2 and TgDHHC7 (multipass integral membrane rhoptry proteins). (B) IFA showing endogenously tagged TgARO-3xHA is distributed along the surface of the entire rhoptry and concentrated at the apex of the organelle. Red: rabbit anti-HA antibody detected by Alexa594-anti-rabbit IgG. Green: mouse anti-ROP2/3/4 antibody detected by Alexa488-anti-mouse IgG. Blue: rat anti-RON11 antibody detected by Alexa350-anti-rat IgG. All scale bars = 5 µm. (C) Western blot showing lower levels of TgARO following promoter replacement. ISP3 serves as a loading control. (D) Untreated TgAROcKO parasites maintain an apical rhoptry bundle (arrow) but also display some dispersed rhoptries (arrowhead). TgARO is visible not only in the apical bundle but also at the apex of each of the individual dispersed rhoptries. (E) Western blot showing knockdown of TgARO after 48 hours of growth with Atc. ISP3 serves as a loading control. (F) Following Atc treatment, cells are depleted of TgARO and the apical bundle of rhoptries is completely lost with the rhoptries scattered throughout the cytosol. (G) Parasites depleted of TgARO encounter a complete block in invasion. Parental or TgAROcKO parasites were grown for 48 hours−/+Atc and then allowed to invade into fresh host cells for one hour. Following depletion of TgARO, parasites show a nearly complete block in host penetration (asterisk, p-value<0.001). A corresponding increase in attached, uninvaded parasites is not seen (blue bars). A minor decrease in penetration is also seen for untreated TgAROcKO, likely due to the lower levels of TgARO expressed in this strain relative to the parental line. (H) Parasites depleted of TgARO cannot form plaques in fibroblast monolayers. Parental or TgAROcKO parasites were grown 48 hours−/+Atc and then infected into fresh fibroblast monolayers at an infective dose of 200 parasites per well and allowed to incubate for nine days. TgAROcKO parasites are unable to form plaques in the presence of Atc, even at an infective dose of 20,000 parasites per well.

TgARO (TGME49_261440) is an attractive candidate substrate for TgDHHC7 by virtue of its palmitoylation-dependent recruitment to the rhoptries and its homology to yeast Vac8p, the substrate of the TgDHHC7 homolog Pfa3 [18]. In keeping with a potential role in apical tethering during late rhoptry maturation, expression timing of TgARO lags behind other rhoptry proteins (including TgDHHC7) by about one hour (Figure 7A). To explore the possibility that TgARO is involved in rhoptry tethering, we first incorporated a 3xHA epitope tag at the endogenous TgARO locus (TgARO-3xHA). As previously reported, TgARO was observed on the entire surface of the rhoptry [18]; however, we also noted that the protein is often concentrated at the apex of the organelle, consistent with a role in apical tethering (Figure 7B).

**Figure 8:**
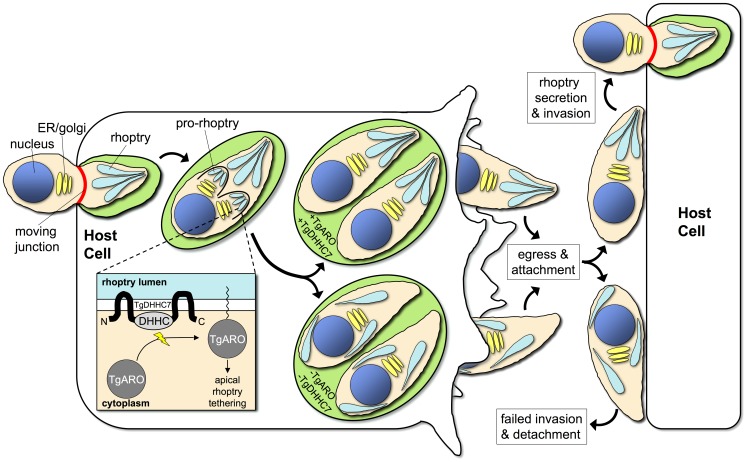
Model of TgDHHC7 and TgARO function in rhoptry biogenesis. TgDHHC7 is positioned in the rhoptry membrane with the catalytic DHHC-CRD domain in the cytosol where it recruits TgARO to the cytosolic face of the rhoptries by palmitoylation. Rhoptry biogenesis occurs *de novo* during each round of parasite replication and recruitment of TgARO by TgDHHC7 during this process facilitates apical rhoptry docking. The failure to recruit TgARO to the rhoptries following knockdown of TgDHHC7 or direct knockdown of TgARO results in the synthesis of rhoptries that are morphologically intact with respect to ultrastructure and cargo, but scattered throughout the parasite cytosol. Without proper tethering at the cell apex, these rhoptries are unable to secrete their contents. While egress from one host cell and attachment to the next occurs normally, the loss of functional rhoptries results in a block in penetration (likely due to a failure to inject rhoptry neck proteins and form a moving junction) and subsequent detachment from the host cell.

To assess TgARO function, we next generated a conditional knockdown by promoter replacement in the TgARO-3xHA line (TgAROcKO). The TgAROcKO strain showed lower levels of basal TgARO expression (Figure 7C) although this decrease was less dramatic than that observed in the TgDHHC7cKO strain (compare with Figure 2A). IFA analysis of untreated TgAROcKO parasites revealed an intact apical bundle of rhoptries (arrow, Figure 7D) as well as some rhoptry dispersion (arrowhead, Figure 7D), similar to TgDHHC7cKO parasites. Culture of TgAROcKO parasites in the presence of Atc for 48 hours dramatically depleted TgARO levels (Figure 7E). Consistent with a role in tethering, knockdown of TgARO was found to recapitulate the phenotype observed upon depletion of TgDHHC7, including the complete loss of apical rhoptry tethering, a major block in host invasion and a failure to form plaques in the presence of Atc (Figure 7F–H). Interestingly, TgARO is still clearly visible at the apex of dispersed rhoptries in untreated TgAROcKO parasites (arrowhead, Figure 7D), suggesting that a threshold concentration of TgARO must be reached to achieve tethering. Collectively, these results demonstrate TgARO is also required for apical rhoptry tethering and strongly suggest that TgARO is recruited to the rhoptry surface by TgDHHC7 to facilitate this process (Figure 8).

A possible role for TgARO in membrane fusion during rhoptry exocytosis is suggested by virtue of its homology to Vac8p, which interacts with the yeast cis-SNARE complex and is required for homotypic vacuole fusion. Palmitoylation of Vac8p by Pfa3 anchors the protein in the vacuole membrane and corresponds with its release from the cis-SNARE complex [43]. In the absence of Vac8p, vacuole docking and formation of trans-SNARE pairs proceeds, but fusion cannot occur [26]. To evaluate the importance of palmitoylation for TgARO function, we attempted to complement the TgDHHC7cKO and TgAROcKO strains with a version of TgARO engineered to target to the rhoptry surface in a palmitoylation-independent fashion. This was accomplished by fusing TgARO (lacking the palmitoylated N-terminal cysteines critical for rhoptry targeting) to the C-terminus of a non-functional version of TgDHHC7 (Figure S7A–B). While this chimeric protein did target to the rhoptries, it was unable to rescue knockdown of either endogenous TgDHHC7 (Figure S7C) or TgARO (data not shown), indicating that more is required to facilitate apical rhoptry tethering than simple localization to the rhoptry surface. A similar experiment with Vac8p also failed to rescue homotypic vacuole fusion in *S. cerevisiae*, indicating that palmitoylation may confer functionality beyond targeted membrane recruitment [44].

Armadillo repeats are comprised of highly variable, ∼42 residue motifs, each composing multiple alpha helices [45]. Tandem repeats of this motif are known to form a superhelix structure that generally functions to promote protein-protein interactions [46]. Thus, understanding the precise mechanism of apical rhoptry tethering will be aided by the identification of TgARO interacting partners. One possibility raised by homology to Vac8p is that TgARO may function to dock the rhoptry neck in a pre-fusion state at the parasite apex through interactions with membrane fusion machinery, priming these organelles for rapid exocytosis during host invasion. A docked, pre-fusion arrangement is also observed in trichocyst secretory organelles harbored by ciliates that are reminiscent of rhoptries in their morphology, regulated secretion and docking at the cell cortex (though not exclusively at the apex) [31]. A further similarity is the presence of a “rosette” of integral plasma membrane proteins at the docking site of both trichocysts and rhoptries, likely representing the machinery involved in docking and/or fusion [47], [48]. Interestingly, the armadillo-repeat containing protein Nd9p has been shown to play a key role in exocytosis of trichocysts in *Paramecium tetraurelia* [49]. While Nd9p contains a series of armadillo-repeats, it does not possess the N-terminal signal for myristoylation and palmitoylation present in TgARO and Vac8p. Furthermore, while Nd9p is required for rosette formation and exocytosis, it does not appear to play a role in positioning trichocysts at the cell cortex, indicating clear differences from the function of TgARO [49], [50].

The fact that TgARO is recruited to the rhoptry surface in a palmitoylation-dependent fashion and that knockdown of either TgDHHC7 or TgARO produces the same phenotype strongly suggests that TgARO is a target for TgDHHC7 (inset, Figure 8). TgARO is the only protein known to be palmitoylated on the rhoptry surface and it will be interesting to determine if additional substrates exist for TgDHHC7 that mediate other functionalities which may be masked by the severity of the rhoptry dispersion phenotype. Additionally, armadillo-repeat proteins (including Vac8p) often serve multiple, independent functions and thus TgARO may have other cellular roles beyond rhoptry tethering [45]. Interestingly, a calcium-dependent phosphorylation event was detected at serine 33 of TgARO, suggesting a possible role for calcium signaling in rhoptry docking and release [51]. However, while calcium signaling is a well-characterized mechanism for controlling microneme secretion, it is not known to play a role in rhoptry regulation [52]. Further work investigating the biological relevance of this modification will clarify its significance to rhoptry tethering or other TgARO functions.

Remarkably, loss of TgDHHC7 or TgARO compromises rhoptry localization and function but not formation, unlike previously reported mutants that abolish rhoptries by impacting secretory traffic destined for these organelles [10], [11]. More importantly, these established secretory mutants ablate both rhoptries and micronemes while depletion of TgDHHC7 or TgARO produces rhoptry-specific defects, providing the first clear demonstration that rhoptries are necessary for invasion but not egress. Together, these findings reveal that PATs play critical roles in unique apicomplexan biology that is foundational to host-parasite interactions and the pathogenesis of these organisms.

## Materials and Methods

### Ethics statement

Antibodies were raised in rats under the guidelines of the Animal Welfare Act and the PHS Policy on Humane Care and Use of Laboratory Animals. Specific details of our protocol were approved by the UCLA Animal Research Committee.

### *Toxoplasma* and host cell culture

*T. gondii* RHΔ*hpt* strain and modified strains were maintained in confluent monolayers of human foreskin fibroblast (HFF) host cells as previously described [53].

### Light microscopy and image processing

Fixation and immunofluorescence staining of *T. gondii* were carried out as previously described [54]. All cells imaged in this study were fixed with 3.5% formaldehyde in PBS. Image stacks were collected at z-increments of 0.2 µm with an AxioCam MRm CCD camera and AxioVision software on an Axio Imager.Z1 microscope (Zeiss) using a 100× oil immersion objective. Deconvolved images were generated using manufacturer specified point-spread functions and displayed as maximum intensity projections.

### Electron microscopy

*T. gondii* strains were cultured −/+1.5 µg/ml Atc for 24 hours, then infected into fresh HFF monolayers and allowed to grow for 24 hours−/+1.5 µg/ml Atc. The cells were then fixed in 2.5% glutaraldehyde (Electron Microscopy Sciences, Hatfield, PA) in 0.1 M sodium cacodylate buffer (pH 7.4) for 1 hr at room temperature and processed as described [55] before examination with a Philips CM120 EM (Eindhoven, the Netherlands) at 80 kV.

### Antibodies and fluorescent fusion constructs

The following *Toxoplasma* primary antibodies were used in IFA or Western blot: rabbit anti-ROP13 [56], polyclonal rat anti-RON11 (see below and Figure S2), mouse anti-IMC1 mAb 45.15 [57], rabbit anti-RON2 [35], rabbit anti-RON4 [37], mouse mAb anti-ROP2/3/4 [58], rabbit anti-SAG1 [59], polyclonal mouse anti-ISP3 [15], mouse anti-F1-ATPase beta subunit mAb 5F4 (Bradley, unpublished), polyclonal mouse anti-DrpB [11], rabbit anti-proROP4 UVT70 [33], mouse anti-MIC2 mAb T3 4A11 [60], mouse anti-ROP1 mAb TG49 [61], and polyclonal mouse anti-RON5C [62]. Hemagglutinin (HA) epitope was detected with mouse mAb HA.11 (Covance), rabbit polyclonal anti-HA (Invitrogen) or rat mAb 3F10 (Roche). FLAG epitope tags were detected with mouse anti-FLAG mAb M2 (Sigma) or rat anti-DYKDDDDK mAb (Stratagene).

For generation of RON11 anti-sera, a portion of RON11 coding sequence comprising the C-terminal 219 residues was cloned from *Toxoplasma* cDNA (primers P1/P2, Table S2) into the expression vector pET101/D-TOPO (Invitrogen). The resulting plasmid was transformed into *E. coli* BL-21DE3 cells, recombinant protein was expressed by 1 mM IPTG induction, purified over Ni-NTA agarose (Qiagen), and injected into a rat for anti-sera production.

For detection of GRASP55, the pGRASP55-YFP [63] plasmid was transfected into parasites allowing for transient expression of the GRASP55-YFP fusion.

### Candidate PAT filtering by expression profiling

The expression timing of 18 *Toxoplasma* genes predicted to encode proteins containing a DHHC-CRD domain (Table S1) was compared to the periodic expression pattern of the known IMC and rhoptry proteins IMC1 and ROP1 [64] and putative PATs were filtered on the basis of their similar to the rhoptry/IMC expression signature of these genes.

### Generation of endogenous epitope tags

For endogenous tagging of TgDHHC7, TgDHHC14 and TgARO, the endogenous tagging vector p3xHA.LIC.DHFR [65] was first modified to replace the DHFR selectable marker cassette with a chloramphenicol acetyl-transferase (CAT) selectable marker between the restriction sites *HindIII*/*XbaI* resulting in the plasmid p3xHA.LIC.CAT. A portion of the genomic locus of each gene up to but not including the stop codon was PCR amplified from *Toxoplasma* genomic DNA (TgDHHC7: P3/P4; TgDHHC14: P5/P6; TgARO: P7/P8) and inserted into p3xHA.LIC.CAT by ligation-independent cloning [66] to generate the vectors pTgDHHC7-3xHA.LIC.CAT, pTgDHHC14-3xHA.LIC.CAT and pTgARO-3xHA.LIC.CAT. These plasmids were linearized with *ApaI*, *MfeI* or *NheI*, respectively, and transfected into the TATiΔ*TgKu80* parasite line [67]. Following selection with chloramphenicol, parasites were cloned by limiting dilution and a clone expressing the tagged protein of interest was isolated and designated TgDHHC7-3xHA, TgDHHC14-3xHA or TgARO-3xHA.

### Generation and complementation of TgDHHC7cKO and TgAROcKO parasites

For direct replacement of the TgDHHC7 or TgARO promoter with the conditional TetOSAG4 promoter by homologous recombination, 5′ (TgDHHC7: P9/P10; TgARO: P13/P14) and 3′ (TgDHHC7: P11/P12; TgARO: P15/P16) regions flanking the promoter were PCR amplified from *Toxoplasma* genomic DNA and cloned into the vector pDT7S4myc [68] between *NdeI* and *BglII*/*AvrII* sites, respectively. The resulting vectors, pTS4-TgDHHC7-DHFR and pTS4-TgARO-DHFR, were linearized with *ApaI* and transfected into TgDHHC7-3xHA or TgARO-3xHA parasites. Following selection with 1 µM pyrimethamine, parasites were cloned by limiting dilution and genomic DNA from individual clones was analyzed by PCR for promoter replacement (TgDHHC7: P17/P18; TgARO:P17/P19). A clone that had undergone the intended recombination event was designated TgDHHC7cKO or TgAROcKO.

For expression of complementing copies of TgDHHC7, the RON5 promoter was PCR amplified from *Toxoplasma* genomic DNA (primers P20/P21) and inserted into the UPRT targeting vector pUPRT-HA [69] between *SpeI* and *BamHI* by blunting both the digested vector and PCR amplicon. A FLAG epitope tag version of this vector was generated by PCR amplifying the 3′ UTR with a forward primer encoding the FLAG epitope sequence (primers P22/P23) and inserting this amplicon between *NotI*/*EcoRV*, replacing the inframe fusion to a C-terminal HA tag with a FLAG tag (pUPRT-FLAG). To complement TgDHHC7cKO parasites, the TgDHHC7 coding sequence was PCR amplified from *Toxoplasma* cDNA (primers P11/P24) and inserted into this vector between *BglII* and *NotI* to generate the vectors pUPRTKO-TgDHHC7-HA/FLAG. The vectors were linearized with *NruI* and transfected into wild-type (to assess targeting) or TgDHHC7cKO parasites followed by selection with 5 µg/ml 5-fluorodeoxyuridine to facilitate targeted replacement of the UPRT locus [70]. For complementation with mutant versions of TgDHHC7, the TgDHHC7 coding sequence was cloned into pJet1.2 (Fermentas) and PCR-based mutagenesis was carried out as previously described [15] (primers P25/P26). Full length and N-terminally truncated (primers P27/P24 and P28/P24) versions of TgDHHC7 encoding the C371S mutation were expressed in TgDHHC7cKO parasites as described above.

### Palmitoylation-independent targeting of TgARO to the rhoptry surface

For targeting of TgARO to the rhoptry surface independent of palmitoylation, the TgARO coding sequence lacking the first six codons that encode the relevant cysteines (P29/P30) and inserted into the vectors pUPRTKO-TgDHHC7-C371S_Δ1–162_-HA/FLAG at *NotI* to create the vectors pTgDHHC7-C371S_Δ1–162_-TgARO_Δ1–6_-HA/FLAG. As a control, the N-terminally truncated TgARO coding sequence was cloned (P31/P30) into pUPRTKO-TgDHHC7-C371S_Δ1–162_-HA/FLAG between *BglII* and *NotI* to generate the vectors pUPRTKO-TgARO_Δ1–6_-HA/FLAG. Each vector was linearized with *NruI* and transfected into wild-type (to assess targeting), TgDHHC7cKO and TgAROcKO parasites followed by selection with 5 µg/ml 5-fluorodeoxyuridine to facilitate targeted replacement of the UPRT locus.

### Generation of TgDHHC7 C-terminal truncations

For TgDHHC7 C-terminal truncations, TgDHHC7 coding sequence under the control of the RON5 promoter was amplified from the vector pUPRTKO-TgDHHC7-WT-FLAG using the indicated primer pairs (FL: P32/P24, Δ511–537: P32/P33, Δ489–537: P32/P34, Δ474–537: P32/P35) and inserted between *Acc65I* and *NotI* in the expression vector pNotI-HA to generate full length and truncated TgDHHC7 expression vectors with a C-terminal fusion to an HA epitope tag. Vectors were linearized with *Acc65I*, transfected into parasites and grown in media containing 50 µg/ml mycophenolic acid and 50 µg/ml xanthine to select for stable integration of the vector.

### Mass spectrometry

Excised gel slices containing TgDHHC7 were digested with trypsin, fractionated online using a C18 reversed phase column, and analyzed by MS/MS on a Thermofisher LTQ-Orbitrap XL as previously described [71], [72]. MS/MS spectra were subsequently analyzed using the ProLuCID and DTASelect algorithms [73], [74].

### Gliding motility assays

Motility assays were performed as previously described [75]. Briefly, parasites were grown 72 hours−/+1.5 µg/ml Atc, monolayers were washed with PBS and intracellular parasites were collected by scrapping and passage through a 27-gauge needle. Equivalent parasite numbers were allowed to glide on FBS-coated glass chamber slides for 30 min before formaldehyde fixation and processing for IFA with rabbit anti-SAG1.

### Plaque assays

Parasites were grown 48 hrs−/+1.5 µg/ml Atc, syringe lysed and infected into 6-well dishes containing fresh, confluent HFF monolayers −/+ Atc. Cultures were allowed to grow nine days before fixation with methanol followed by staining with crystal violet.

### Invasion, evacuole and egress assays

Invasion assays were performed as previously described [76]. Briefly, parasites were grown 72 hrs−/+1.5 µg/ml Atc, monolayers were washed with PBS and intracellular parasites were collected by scrapping and passage through a 27-gauge needle. Equivalent parasite numbers were resuspended in pre-warmed media and allowed to infect HFF monolayers on coverslips for one hour. Monolayers were then washed, fixed with EM-grade 3.7% formaldehyde/PBS (Biosciences, Inc.), blocked with PBS/3%BSA for 30 min and incubated with rabbit anti-SAG1 diluted in PBS/3%BSA for 1 hr. After washing, samples were permeabilized in PBS/3%BSA/0.1% Triton X-100 for 30 min and then incubated with mAb 5F4 diluted in PBS/3%BSA for one hour. Following incubation with secondary antibodies, samples were examined by fluorescence microscopy and parasites were scored as invaded (SAG1−, 5F4+) or attached (SAG1+, 5F4+). Invasion assays were performed in triplicate, five fields were counted on each replicate coverslip and the average number of invaded and attached parasites per field was calculated.

For evacuole assays, parasites were grown −/+1.5 µg/ml Atc for 36 hours to allow large vacuoles to form and intracellular parasites were collected by scrapping and passage through a 27-gauge needle. Evacuole assays were then performed as previously described [77]. The number of evacuoles were counted across five fields per coverslip on three independent coverslips per sample and the average number per field was calculated.

Egress assays were performed as previously described [78]. Briefly, parasites were grown −/+1.5 µg/ml Atc for 24 hours, then infected into fresh HFF monolayers on coverslips and allowed to grow an additional 36 hours −/+1.5 µg/ml Atc. Coverslips were then washed with PBS and incubated in 1 µM calcium ionophore A23187 (Sigma) diluted in Hanks Balances Salts Solution at 37° before being fixed in methanol and processed for IFA with rabbit anti-SAG1. At least 100 vacuoles per coverslip were counted across five fields on three independent coverslips per sample and scored as egressed or not egressed. For each of the above assays, experiments were repeated at least twice and values from a representative experiment are shown as the mean ± SD.

## Supporting Information

Figure S1 Candidate PAT filtering by cell cycle expression profiling. Notably, IMC and rhoptry genes display a similar pattern with expression levels peaking in a narrow 1-hour window during daughter bud formation (see Figure 1A).
(TIF)

Figure S2 Identification, localization and initial characterization of the novel rhoptry neck protein RON11. Our earlier proteomic analysis of *Toxoplasma* rhoptries identified several putative rhoptry proteins that have not yet been confirmed, including the gene TGME49_230350 (formerly annotated as TGTWINSCAN_5713) [54]. (A) The TGME49_230350 gene model, which we confirmed by cDNA sequencing (GenBank accession number KC347564), predicts a 1254 residue protein with at least four predicted transmembrane domains (black) and a C-terminal EF hand domain (green), suggesting a role for this protein in binding calcium and/or sensing calcium fluctuation. The protein is conserved across the Apicomplexa, including orthologs in *Plasmodium spp*. [54]. To localize TGME49_230350, we raised rat anti-sera against a recombinantly expressed portion of the protein corresponding to the C-terminal 219 residues (bracket). (B–C) IFA analysis of parasites using the rat anti-sera raised against TGME49_230350. (B) The antibody was found to stain the neck portion of the rhoptry organelle, as shown by co-localization with RON2, and the protein was thus named RON11. Red: anti-RON11 antibody detected by Alexa594-anti-rat IgG. Green: rabbit anti-RON2 antibody detected by Alexa488-anti-rabbit IgG. Scale bar = 5 µm. (C) Early invasion IFA assay showing a parasite in the act of host penetration. Unlike RON4, RON11 does not relocalize from the rhoptry neck (arrowheads) to the moving junction (arrows) during host invasion. Green: anti-RON11 antibody detected by Alexa488-anti-rat IgG. Red: rabbit anti-RON4 antibody detected by Alexa594-anti-rabbit IgG. (D) Western blot using the rat-anti-RON11 antibody detects a major band at ∼130 kD in agreement with the predicted size of the protein. In addition, a minor band is detected at >170 kD. The large size of this minor band may represent a size shift due to post-translational modification or multimerization of the protein that fails to dissociate during SDS-PAGE.
(TIF)

Figure S3 Determination of the correct TgDHHC7 start codon. (A) TgDHHC7 protein sequence based on cDNA sequencing and RNAseq analysis. Three start codons are possible (boxed) in the first exon with corresponding protein masses predicted at 60, 43, and 38 kD. (B) Western blot analysis of our endogenously tagged strain (TgDHHC7-3xHA) shows a single band at ∼45 kD. Taking into account the size of the 3xHA tag (4.6 kD), this indicates the endogenous protein migrates at ∼40 kD, most consistent with the second start codon. (C) To resolve ambiguity regarding the true start codon, we first performed MS/MS analysis on purified TgDHHC7-3xHA. TgDHHC7-3xHA was immunoprecipitated from parasite lysates and resolved by SDS-PAGE. The resulting Coomassie stained band (arrow) was cut from the gel, digested with trypsin and analyzed by MS/MS. The TgDHHC7 peptides discovered by MS/MS (shown in red in A) eliminate the possibility of M3 as the start codon. (B) To determine whether M1 or M2 is the correct start codon, we then expressed TgDHHC7 cDNAs starting at either the M1 or M2 methonine with a C-terminal 1xHA tag and compared migration of the resulting proteins to the endogenously tagged TgDHHC7-3xHA. While migration of the endogenously tagged protein is most consistent with the predicted size of M2 as the start codon, a TgDHHC7 cDNA beginning at M2 (TgDHHC7_M2_-1xHA) was found to migrate at ∼30 kD, 15 kD smaller than predicted from primary sequence, indicating that M1 is the correct start codon and that TgDHHC7 migrates faster than expected, as is commonly observed for multi-pass transmembrane proteins [79]. Indeed, a TgDHHC7 cDNA beginning at M1 (TgDHHC7_M1_-1xHA) was found to migrate slightly faster than TgDHHC7-3xHA, consistent with the 3.3 kD difference in size due to the 1xHA vs 3xHA tag in these two proteins. (D) Table summarizing the expected and observed sizes of the various forms of TgDHHC7 examined.
(TIF)

Figure S4 Alignment of TgDHHC7 sequence with orthologs in other species identified by BLAST. The highly conserved DHHC (red box), predicted transmembrane domains (black underline), PaCCT motif (red underline) and a C-terminal conserved region (blue underline) are indicated. Species abbreviations and accession numbers: *Saccharomyces cerevisiae* (Pfa3), NP_014073; *Plasmodium falciparum* (Pf), XP_001351838; *Plasmodium vivax* (Pv), XP_001613674; *Theileria annulata* (Ta), XP_952273; *Babesia bovis* (Bb), XP_001611639; *Cryptosporidium muris* (Cm), XP_002141787; *Eimeria tenella* (Et), AET50820; *Toxoplasma gondii* (Tg), AFW99807.
(TIF)

Figure S5 (A) Analysis of proteolytic processing of rhoptry proteins following knockdown of TgDHHC7. TgDHHC7cKO parasites were grown −/+ Atc for 48 hours before harvesting parasites. Processing of rhoptry body proteins ROP1 and ROP13 and the rhoptry neck protein RON5C was assessed by Western blot. No difference in the ratio of pro to mature forms of these proteins was observed, indicating proteolytic processing of rhoptry contents proceeds normally in dispersed rhoptries. (B) DrpB localization and dynamics are unaffected by knockdown of TgDHHC7. IFA of untreated parental parasites and TgDHHC7cKO parasites following growth with Atc for 60 hours. Rhoptries are scattered throughout the cell in TgDHHC7cKO parasites following Atc treatment, as assessed by staining for ROP13 and RON11. However, no change was observed in the signal strength or localization pattern of the dynamin-like protein DrpB. Red: anti-DrpB antibody detected by Alexa594-anti-mouse IgG. Green: anti-ROP13 antibody detected by Alexa488-anti-rabbit IgG. Blue: anti-RON11 antibody detected by Alexa350-anti-rat IgG. Scale bars = 5 µm. (C–D) Minor abnormalities inTgDHHC7cKO parasites observed by TEM. (C) Parasites lacking TgDHHC7 were sometimes (<9% of TEM sections) observed to contain amylopectin granules (arrows), which may be a sign of stress. (D) Multi-membranous bodies (arrow) of unclear origin were sometimes (<7% of TEM sections) observed in parasites lacking TgDHHC7.
(TIF)

Figure S6 Microneme biosynthesis and parasite gliding motility are unaffected by TgDHHC7 knockdown. (A) IFA analysis of micronemes in parental and TgDHHC7cKO parasites. Parental parasites were grown − Atc while TgDHHC7cKO parasites were grown 60 hours + Atc prior to fixation and processing. Micronemes are unaffected upon knockdown of TgDHHC7, as assessed by staining for the microneme protein MIC2. In contrast, rhoptries are scattered throughout the cell in TgDHHC7cKO parasites following Atc treatment, as assessed by staining for ROP13 and RON11. Red: anti-MIC2 antibody detected by Alexa594-anti-mouse IgG. Green: anti-ROP13 antibody detected by Alexa488-anti-rabbit IgG. Blue: anti-RON11 antibody detected by Alexa350-anti-rat IgG. Scale bars = 5 µm. (B) Gliding motility, which requires secretion of micronemal adhesions, was assayed as a measure of microneme functionality. No difference was observed in the frequency or length of SAG1 trails deposited by parental or TgDHHC7cKO parasites −/+ Atc treatment.
(TIF)

Figure S7 Palmitoylation-independent targeting of TgARO to the rhoptry surface is unable to rescue the defects incurred upon knockdown of TgDHHC7 or TgARO. (A) N-terminal truncation of the first six residues of TgARO removes the myristoylation and palmitoylation signals that are critical for rhoptry targeting, resulting in gross mistargeting throughout the cytosol. Red: mouse anti-HA antibody detected by Alexa594-anti-mouse IgG. Green: rabbit anti-ROP13 antibody detected by Alexa488-anti-rabbit. Scale bar = 5 µm. (B) Fusion of this N-terminally truncated version of TgARO to the C-terminus of a C371S mutant form of TgDHHC7 (TgDHHC7-C371S_Δ1–162_) restores targeting of TgARO to the rhoptry surface. Red: anti-HA antibody detected by Alexa594-anti-rabbit IgG. Green: mouse anti-ROP2/3/4 antibody detected by Alexa488-anti-mouse. Scale bar = 5 µm. (C) Complementation of TgDHHC7cKO assessed by plaque assay. Neither the TgARO_Δ1–6_ truncation mutant or the chimeric TgDHHC7-TgARO fusion are able to rescue the defect incurred by the knockdown of endogenous TgDHHC7. These complemented strains still fail to apically tether rhoptries (assessed by IFA, data not shown) and cannot form plaques in the presence of Atc. Similarly, complementation of TgAROcKO parasites with the chimeric TgDHHC7-TgARO fusion also failed to rescue knockdown of TgARO (data not shown).
(TIF)

Movie S1 Rhoptries fail to dock at the cell apex following depletion of TgDHHC7. Deconvolved, serial sections from Figure 2A–C were projected as a three-dimensional image. Rhoptries are bundled and docked at the cell apex in wild-type parasites (Parent, top panel). In untreated TgDHHC7cKO parasites, the majority of rhoptries continue to dock at the apex a form an apical bundle. A few scattered rhoptries are present, presumably due to lower levels of TgDHHC7 basal expression in this strain. Following Atc treatment of TgDHHC7cKO and loss of TgDHHC7, apical bundles of rhoptries are lost with individual rhoptries instead scattered throughout the cell cytosol.
(MOV)

Table S1 DHHC-CRD containing proteins encoded within the *Toxoplasma* genome.
(DOC)

Table S2 Primers used in this study as discussed in the text. Restriction sites and mutated bases are shown in lowercase.
(DOC)
