# Supplementary figures and images for: A Toxoplasma Palmitoyl Acyl Transferase and the Palmitoylated Armadillo Repeat Protein TgARO Govern Apical Rhoptry Tethering and Reveal a Critical Role for the Rhoptries in Host Cell Invasion but Not Egress
Source: PLoS Pathog. 2013 Feb 7;9(2):e1003162. doi: 10.1371/journal.ppat.1003162 (PMC3567180; doi:10.1371/journal.ppat.1003162)

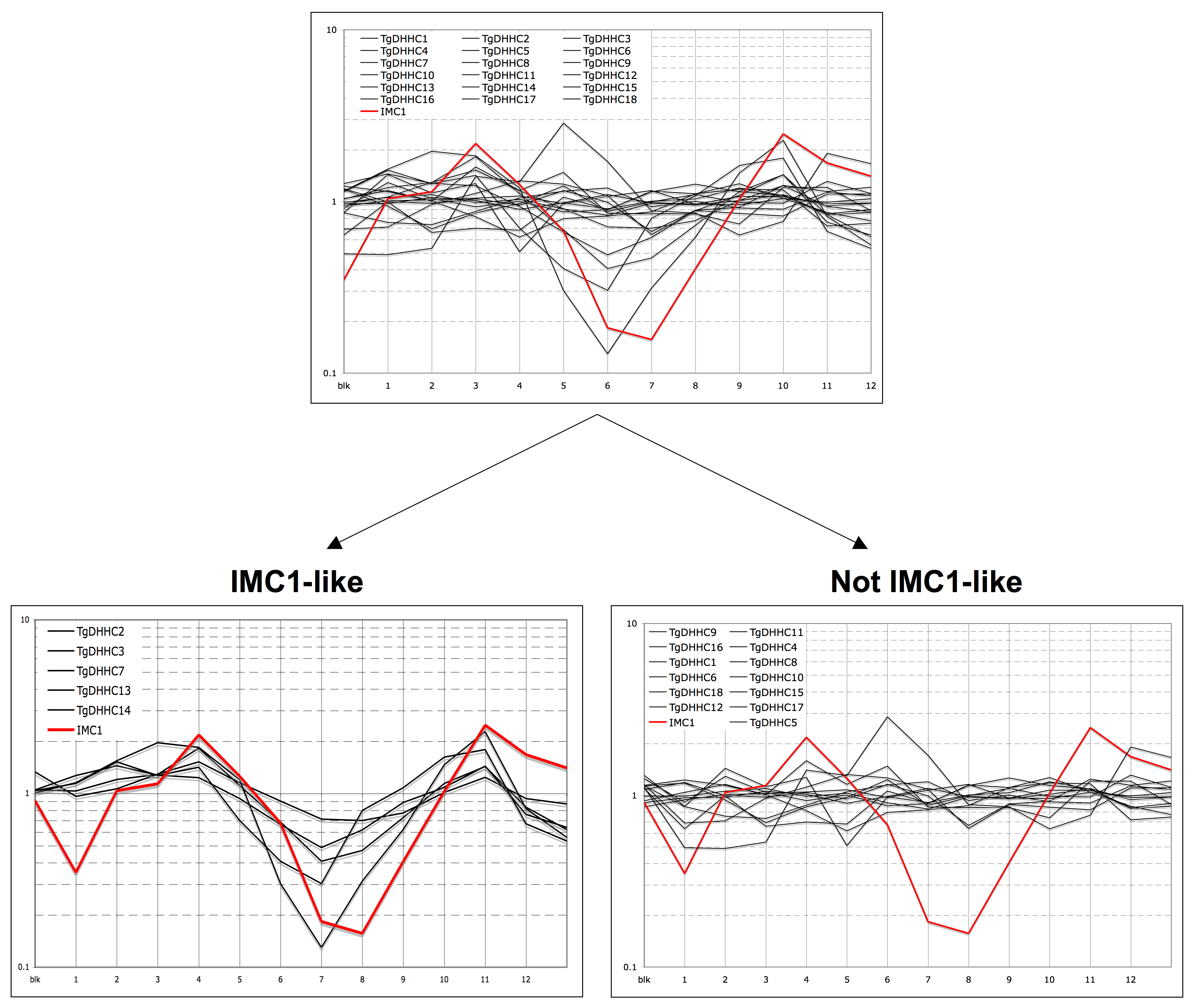

Supplement: Figure S1 — Candidate PAT filtering by cell cycle expression profiling. Notably, IMC and rhoptry genes display a similar pattern with expression levels peaking in a narrow 1-hour window during daughter bud formation (see Figure 1A). (TIF) [file ppat.1003162.s001.tif]

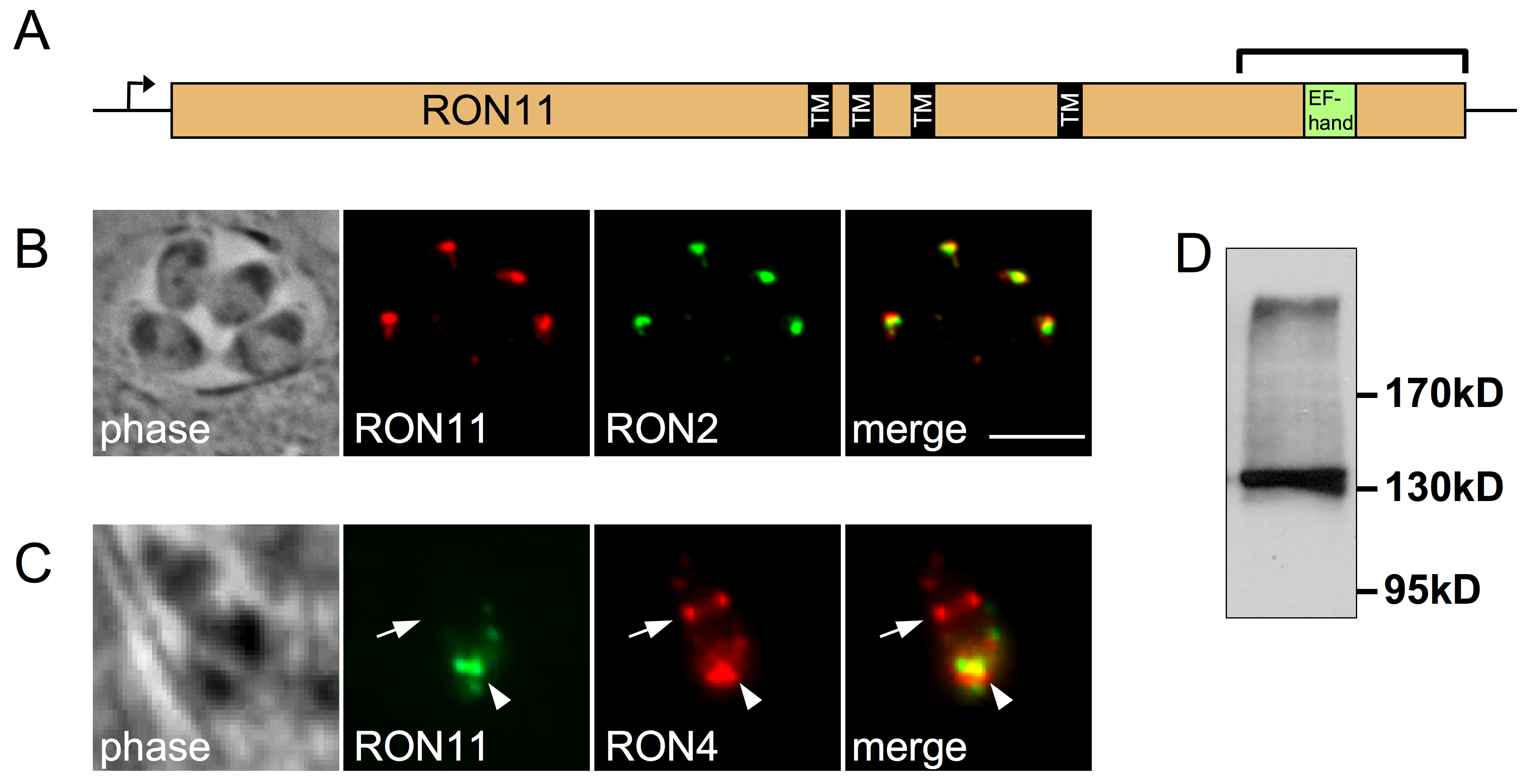

Supplement: Figure S2 — Identification, localization and initial characterization of the novel rhoptry neck protein RON11. Our earlier proteomic analysis of Toxoplasma rhoptries identified several putative rhoptry proteins that have not yet been confirmed, including the gene TGME49_230350 (formerly annotated as TGTWINSCAN_5713) [54]. (A) The TGME49_230350 gene model, which we confirmed by cDNA sequencing (GenBank accession number KC347564), predicts a 1254 residue protein with at least four predicted transmembrane domains (black) and a C-terminal EF hand domain (green), suggesting a role for this protein in binding calcium and/or sensing calcium fluctuation. The protein is conserved across the Apicomplexa, including orthologs in Plasmodium spp. [54]. To localize TGME49_230350, we raised rat anti-sera against a recombinantly expressed portion of the protein corresponding to the C-terminal 219 residues (bracket). (B–C) IFA analysis of parasites using the rat anti-sera raised against TGME49_230350. (B) The antibody was found to stain the neck portion of the rhoptry organelle, as shown by co-localization with RON2, and the protein was thus named RON11. Red: anti-RON11 antibody detected by Alexa594-anti-rat IgG. Green: rabbit anti-RON2 antibody detected by Alexa488-anti-rabbit IgG. Scale bar = 5 µm. (C) Early invasion IFA assay showing a parasite in the act of host penetration. Unlike RON4, RON11 does not relocalize from the rhoptry neck (arrowheads) to the moving junction (arrows) during host invasion. Green: anti-RON11 antibody detected by Alexa488-anti-rat IgG. Red: rabbit anti-RON4 antibody detected by Alexa594-anti-rabbit IgG. (D) Western blot using the rat-anti-RON11 antibody detects a major band at ∼130 kD in agreement with the predicted size of the protein. In addition, a minor band is detected at >170 kD. The large size of this minor band may represent a size shift due to post-translational modification or multimerization of the protein that fails to dissociate during SDS-PAGE. (TIF) [file ppat.1003162.s002.tif]

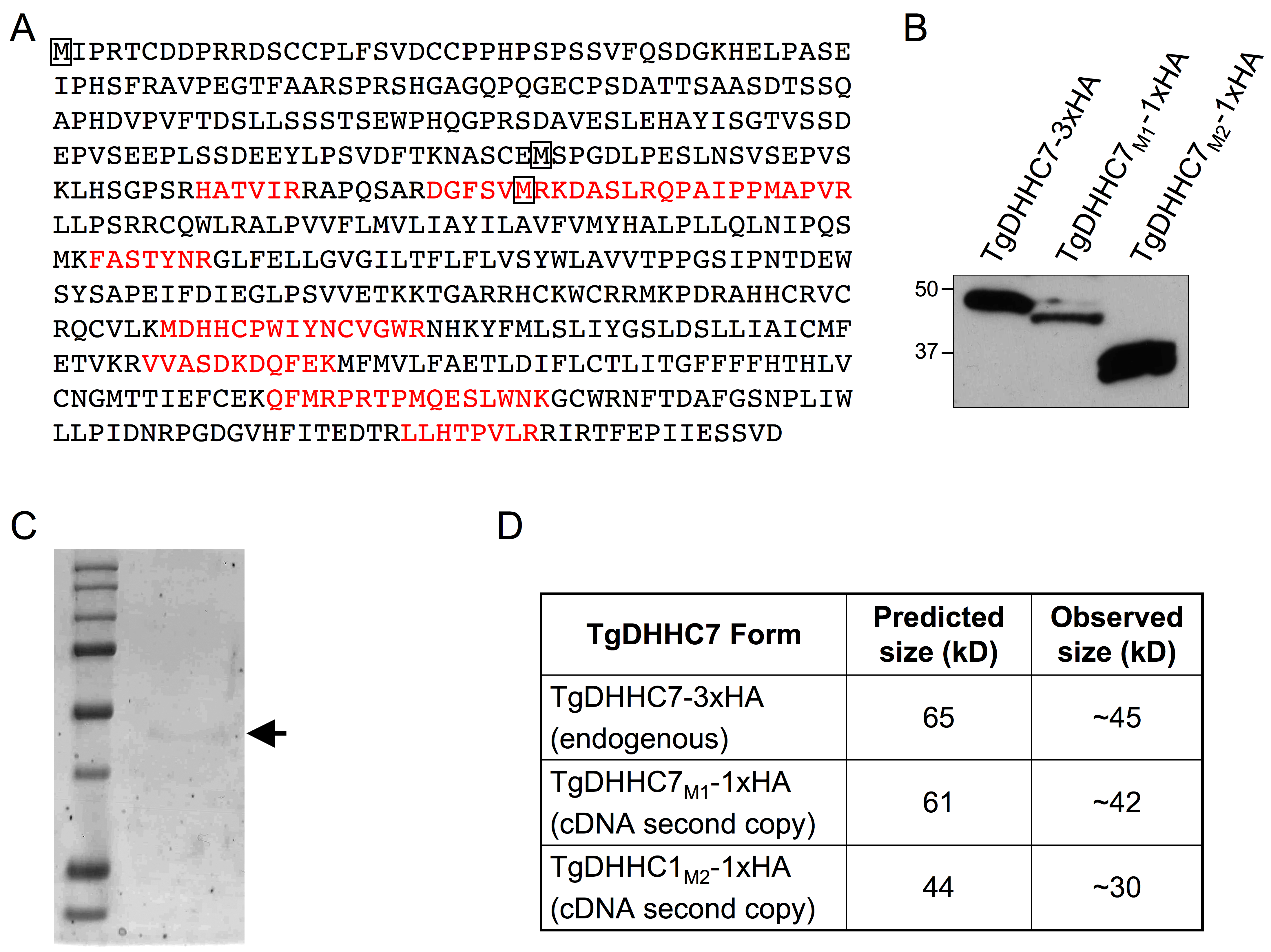

Supplement: Figure S3 — Determination of the correct TgDHHC7 start codon. (A) TgDHHC7 protein sequence based on cDNA sequencing and RNAseq analysis. Three start codons are possible (boxed) in the first exon with corresponding protein masses predicted at 60, 43, and 38 kD. (B) Western blot analysis of our endogenously tagged strain (TgDHHC7-3xHA) shows a single band at ∼45 kD. Taking into account the size of the 3xHA tag (4.6 kD), this indicates the endogenous protein migrates at ∼40 kD, most consistent with the second start codon. (C) To resolve ambiguity regarding the true start codon, we first performed MS/MS analysis on purified TgDHHC7-3xHA. TgDHHC7-3xHA was immunoprecipitated from parasite lysates and resolved by SDS-PAGE. The resulting Coomassie stained band (arrow) was cut from the gel, digested with trypsin and analyzed by MS/MS. The TgDHHC7 peptides discovered by MS/MS (shown in red in A) eliminate the possibility of M3 as the start codon. (B) To determine whether M1 or M2 is the correct start codon, we then expressed TgDHHC7 cDNAs starting at either the M1 or M2 methonine with a C-terminal 1xHA tag and compared migration of the resulting proteins to the endogenously tagged TgDHHC7-3xHA. While migration of the endogenously tagged protein is most consistent with the predicted size of M2 as the start codon, a TgDHHC7 cDNA beginning at M2 (TgDHHC7M2-1xHA) was found to migrate at ∼30 kD, 15 kD smaller than predicted from primary sequence, indicating that M1 is the correct start codon and that TgDHHC7 migrates faster than expected, as is commonly observed for multi-pass transmembrane proteins [79]. Indeed, a TgDHHC7 cDNA beginning at M1 (TgDHHC7M1-1xHA) was found to migrate slightly faster than TgDHHC7-3xHA, consistent with the 3.3 kD difference in size due to the 1xHA vs 3xHA tag in these two proteins. (D) Table summarizing the expected and observed sizes of the various forms of TgDHHC7 examined. (TIF) [file ppat.1003162.s003.tif]

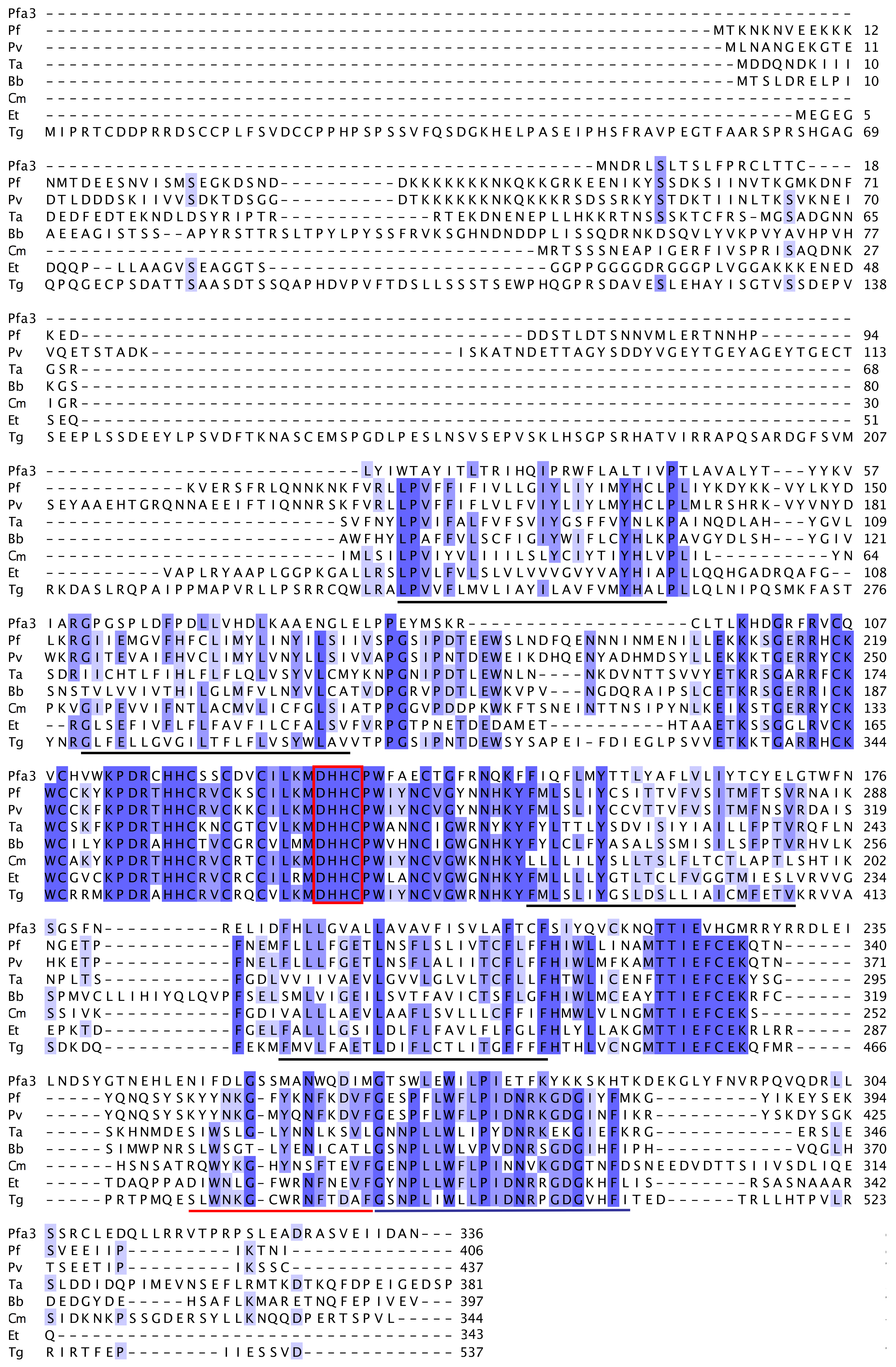

Supplement: Figure S4 — Alignment of TgDHHC7 sequence with orthologs in other species identified by BLAST. The highly conserved DHHC (red box), predicted transmembrane domains (black underline), PaCCT motif (red underline) and a C-terminal conserved region (blue underline) are indicated. Species abbreviations and accession numbers: Saccharomyces cerevisiae (Pfa3), NP_014073; Plasmodium falciparum (Pf), XP_001351838; Plasmodium vivax (Pv), XP_001613674; Theileria annulata (Ta), XP_952273; Babesia bovis (Bb), XP_001611639; Cryptosporidium muris (Cm), XP_002141787; Eimeria tenella (Et), AET50820; Toxoplasma gondii (Tg), AFW99807. (TIF) [file ppat.1003162.s004.tif]

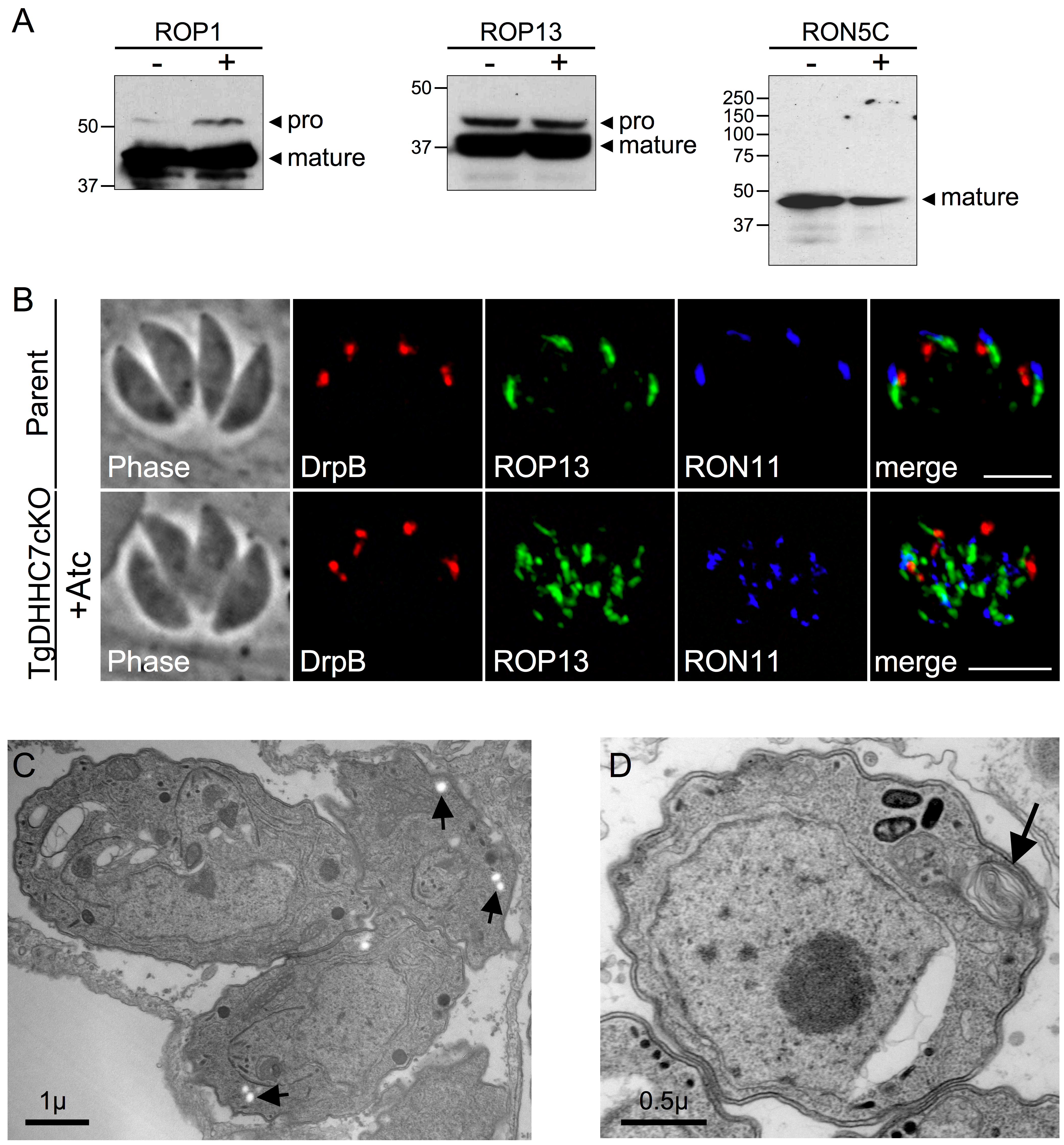

Supplement: Figure S5 — (A) Analysis of proteolytic processing of rhoptry proteins following knockdown of TgDHHC7. TgDHHC7cKO parasites were grown −/+ Atc for 48 hours before harvesting parasites. Processing of rhoptry body proteins ROP1 and ROP13 and the rhoptry neck protein RON5C was assessed by Western blot. No difference in the ratio of pro to mature forms of these proteins was observed, indicating proteolytic processing of rhoptry contents proceeds normally in dispersed rhoptries. (B) DrpB localization and dynamics are unaffected by knockdown of TgDHHC7. IFA of untreated parental parasites and TgDHHC7cKO parasites following growth with Atc for 60 hours. Rhoptries are scattered throughout the cell in TgDHHC7cKO parasites following Atc treatment, as assessed by staining for ROP13 and RON11. However, no change was observed in the signal strength or localization pattern of the dynamin-like protein DrpB. Red: anti-DrpB antibody detected by Alexa594-anti-mouse IgG. Green: anti-ROP13 antibody detected by Alexa488-anti-rabbit IgG. Blue: anti-RON11 antibody detected by Alexa350-anti-rat IgG. Scale bars = 5 µm. (C–D) Minor abnormalities inTgDHHC7cKO parasites observed by TEM. (C) Parasites lacking TgDHHC7 were sometimes (<9% of TEM sections) observed to contain amylopectin granules (arrows), which may be a sign of stress. (D) Multi-membranous bodies (arrow) of unclear origin were sometimes (<7% of TEM sections) observed in parasites lacking TgDHHC7. (TIF) [file ppat.1003162.s005.tif]

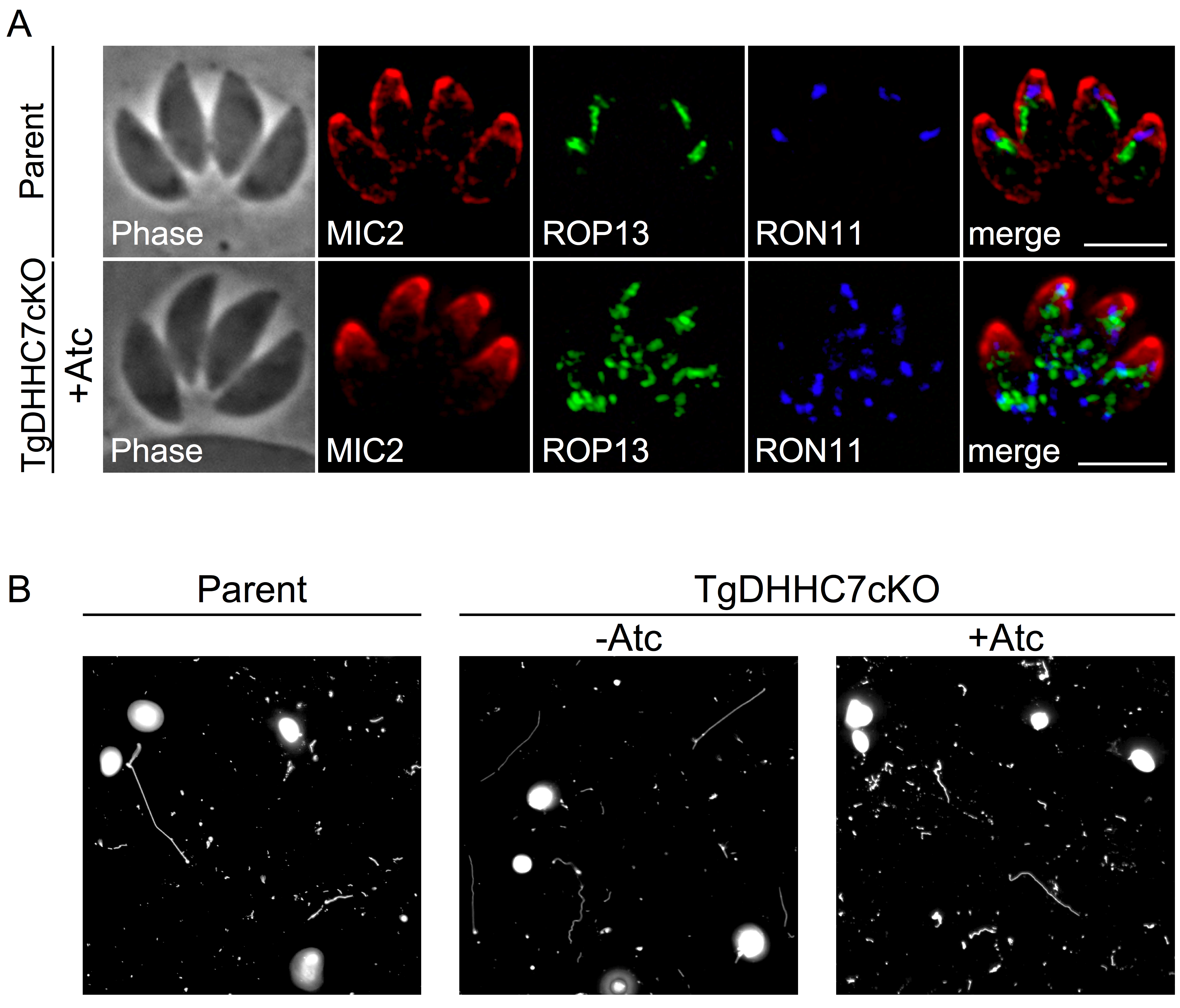

Supplement: Figure S6 — Microneme biosynthesis and parasite gliding motility are unaffected by TgDHHC7 knockdown. (A) IFA analysis of micronemes in parental and TgDHHC7cKO parasites. Parental parasites were grown − Atc while TgDHHC7cKO parasites were grown 60 hours + Atc prior to fixation and processing. Micronemes are unaffected upon knockdown of TgDHHC7, as assessed by staining for the microneme protein MIC2. In contrast, rhoptries are scattered throughout the cell in TgDHHC7cKO parasites following Atc treatment, as assessed by staining for ROP13 and RON11. Red: anti-MIC2 antibody detected by Alexa594-anti-mouse IgG. Green: anti-ROP13 antibody detected by Alexa488-anti-rabbit IgG. Blue: anti-RON11 antibody detected by Alexa350-anti-rat IgG. Scale bars = 5 µm. (B) Gliding motility, which requires secretion of micronemal adhesions, was assayed as a measure of microneme functionality. No difference was observed in the frequency or length of SAG1 trails deposited by parental or TgDHHC7cKO parasites −/+ Atc treatment. (TIF) [file ppat.1003162.s006.tif]

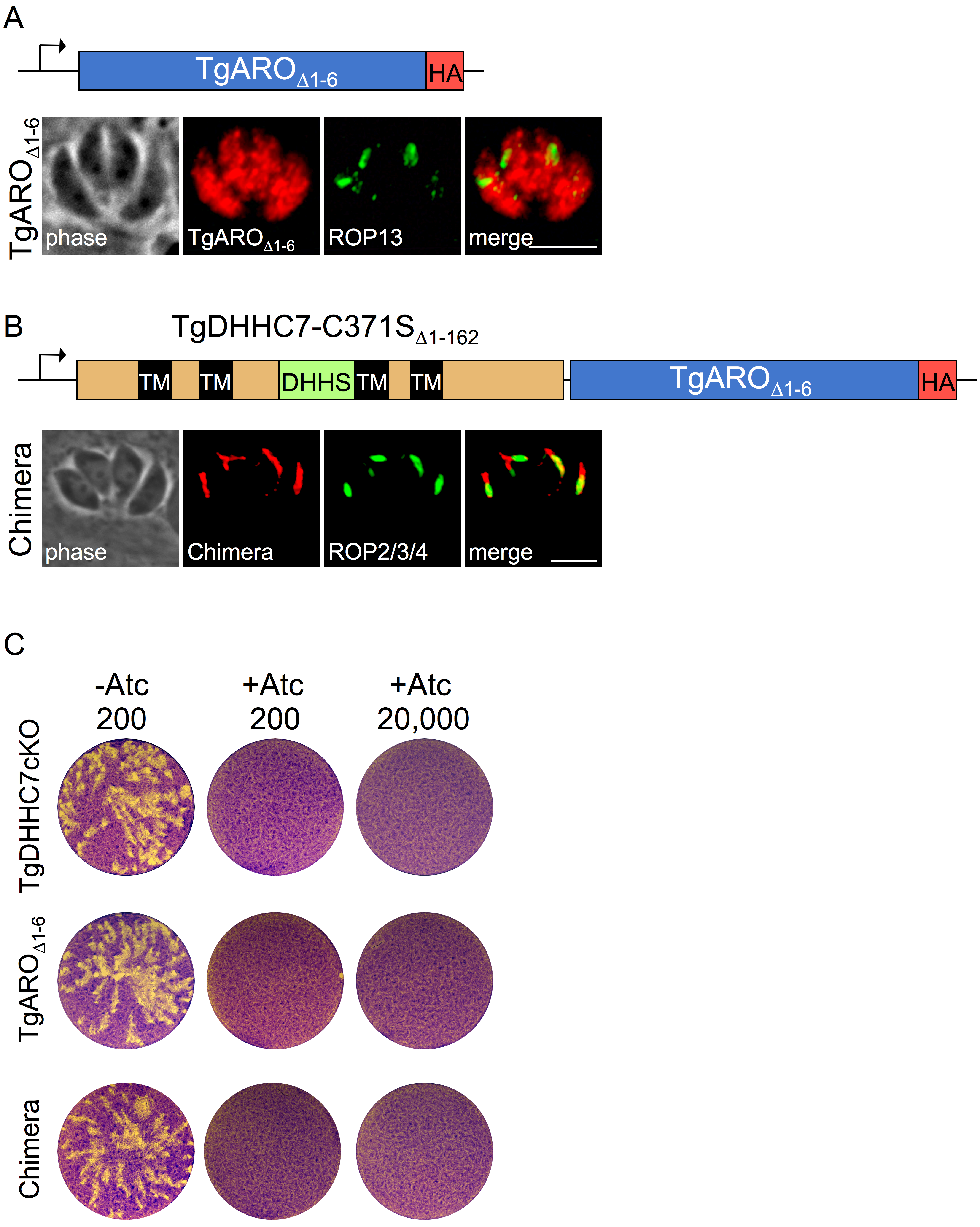

Supplement: Figure S7 — Palmitoylation-independent targeting of TgARO to the rhoptry surface is unable to rescue the defects incurred upon knockdown of TgDHHC7 or TgARO. (A) N-terminal truncation of the first six residues of TgARO removes the myristoylation and palmitoylation signals that are critical for rhoptry targeting, resulting in gross mistargeting throughout the cytosol. Red: mouse anti-HA antibody detected by Alexa594-anti-mouse IgG. Green: rabbit anti-ROP13 antibody detected by Alexa488-anti-rabbit. Scale bar = 5 µm. (B) Fusion of this N-terminally truncated version of TgARO to the C-terminus of a C371S mutant form of TgDHHC7 (TgDHHC7-C371SΔ1–162) restores targeting of TgARO to the rhoptry surface. Red: anti-HA antibody detected by Alexa594-anti-rabbit IgG. Green: mouse anti-ROP2/3/4 antibody detected by Alexa488-anti-mouse. Scale bar = 5 µm. (C) Complementation of TgDHHC7cKO assessed by plaque assay. Neither the TgAROΔ1–6 truncation mutant or the chimeric TgDHHC7-TgARO fusion are able to rescue the defect incurred by the knockdown of endogenous TgDHHC7. These complemented strains still fail to apically tether rhoptries (assessed by IFA, data not shown) and cannot form plaques in the presence of Atc. Similarly, complementation of TgAROcKO parasites with the chimeric TgDHHC7-TgARO fusion also failed to rescue knockdown of TgARO (data not shown). (TIF) [file ppat.1003162.s007.tif]
