# Supplementary material for: A Toxoplasma Palmitoyl Acyl Transferase and the Palmitoylated Armadillo Repeat Protein TgARO Govern Apical Rhoptry Tethering and Reveal a Critical Role for the Rhoptries in Host Cell Invasion but Not Egress
Source: PLoS Pathog. 2013 Feb 7;9(2):e1003162. doi: 10.1371/journal.ppat.1003162 (PMC3567180; doi:10.1371/journal.ppat.1003162)
Supplement: Table S1 — DHHC-CRD containing proteins encoded within the Toxoplasma genome. (DOC) [file ppat.1003162.s009.doc]

**Table S1**

DHHC-CRD containing proteins encoded within the *Toxoplasma* genome.

| Name | ToxoDB Accession Number | Length | Signal Peptide? | TMs | Other Domains? |
| --- | --- | --- | --- | --- | --- |
| TgDHHC1 | TGME49_250870 | 361 | N | 4 | N |
| TgDHHC2 | TGME49_278850 | 361 | N | 4 | N |
| TgDHHC3 | TGME49_217870 | 430 | Y | 4 | N |
| TgDHHC4 | TGME49_213550 | 1362 | N | 4 | N |
| TgDHHC5 | TGME49_224290 | 371 | N | 3 | N |
| TgDHHC6 | TGME49_224310 | 391 | N | 4 | N |
| TgDHHC7 | TGME49_252200 | 537 | N | 4 | N |
| TgDHHC8 | TGME49_255650 | 471 | Y | 6 | N |
| TgDHHC9 | TGME49_269150 | 356 | Y | 4 | N |
| TgDHHC10 | TGME49_301370 | 278 | Y | 4 | N |
| TgDHHC11 | TGME49_284170 | 951 | N | 3 | N |
| TgDHHC12 | TGME49_229160 | 693 | N | 3 | N |
| TgDHHC13 | TGME49_249380 | 466 | N | 4 | N |
| TgDHHC14 | TGME49_293730 | 971 | Y | 2 | Ankyrin repeats |
| TgDHHC15 | TGME49_293220 | 1327 | N | 2 | N |
| TgDHHC16 | TGME49_266940 | 1044 | N | 4 | N |
| TgDHHC17 | TGME49_272320 | 943 | N | 3 | Ankyrin repeats |
| TgDHHC18 | TGME49_246650 | 560 | N | 2 | N |

Relevant information was acquired from ToxoDB (release 8.0). TMs = number of predicted transmembrane domains.
