# Supplementary material for: A Toxoplasma Palmitoyl Acyl Transferase and the Palmitoylated Armadillo Repeat Protein TgARO Govern Apical Rhoptry Tethering and Reveal a Critical Role for the Rhoptries in Host Cell Invasion but Not Egress
Source: PLoS Pathog. 2013 Feb 7;9(2):e1003162. doi: 10.1371/journal.ppat.1003162 (PMC3567180; doi:10.1371/journal.ppat.1003162)
Supplement: Table S2 — Primers used in this study as discussed in the text. Restriction sites and mutated bases are shown in lowercase. (DOC) [file ppat.1003162.s010.doc]

Table S2

| **Table S1. DHHC-contain proteins encoded within the *Toxoplasma* genome. Relevant information was aquired from ToxoDB (release 8.0).**   |  | | | | | | | | --- | --- | --- | --- | --- | --- | --- | | Signal Peptide?TMsProteomics?Other Domains?Name | | | | | | | | TgDHHC1ToxoDB Accession NumberLength | TGME49_250870 | 361 | N | 4 | Y | N | | TgDHHC2 | TGME49_278850 | 361 | N | 4 | Y | N | | TgDHHC3 | TGME49_217870 | 430 | Y | 4 | Y | N | | TgDHHC4 | TGME49_213550 | 1362 | N | 4 | Y | N | | TgDHHC5 | TGME49_224290 | 371 | N | 3 | N | N | | TgDHHC6 | TGME49_224310 | 391 | N | 4 | N | N | | TgDHHC7 | TGME49_252200 | 537 | N | 4 | N | N | | TgDHHC8 | TGME49_255650 | 471 | Y | 6 | Y | N | | TgDHHC9 | TGME49_269150 | 356 | Y | 4 | Y | N | | TgDHHC10 | TGME49_301370 | 278 | Y | 4 | N | N | | TgDHHC11 | TGME49_284170 | 951 | N | 3 | N | N | | TgDHHC12 | TGME49_229160 | 693 | N | 3 | Y | N | | TgDHHC13 | TGME49_249380 | 466 | N | 4 | Y | N | | TgDHHC14 | TGME49_293730 | 971 | Y | 2 | Y | Ankyrin repeats | | TgDHHC15 | TGME49_293220 | 1327 | N | 2 | Y | N | | TgDHHC16 | TGME49_266940 | 1044 | N | 4 | N | N | | TgDHHC17 | TGME49_272320 | 943 | N | 3 | N | Ankyrin repeats | | TgDHHC18 | TGME49_246650 | 560 | N | 2 | N | N | |  |  |  |  |  |  |  | | | | | | | | |
| --- | --- | --- | --- | --- | --- | --- | --- | --- | --- | --- | --- | --- | --- | --- | --- | --- | --- | --- | --- | --- | --- | --- | --- | --- | --- | --- | --- | --- | --- | --- | --- | --- | --- | --- | --- | --- | --- | --- | --- | --- | --- | --- | --- | --- | --- | --- | --- | --- | --- | --- | --- | --- | --- | --- | --- | --- | --- | --- | --- | --- | --- | --- | --- | --- | --- | --- | --- | --- | --- | --- | --- | --- | --- | --- | --- | --- | --- | --- | --- | --- | --- | --- | --- | --- | --- | --- | --- | --- | --- | --- | --- | --- | --- | --- | --- | --- | --- | --- | --- | --- | --- | --- | --- | --- | --- | --- | --- | --- | --- | --- | --- | --- | --- | --- | --- | --- | --- | --- | --- | --- | --- | --- | --- | --- | --- | --- | --- | --- | --- | --- | --- | --- | --- | --- | --- | --- | --- | --- | --- | --- | --- | --- | --- | --- | --- | --- | --- | --- | --- | --- | --- | --- | --- | --- |
|  |  |  |  |  |  |  |  |
|  |  |  |  |  |  |  |  |
|  |  |  |  |  |  |  |  |
|  |  |  |  |  |  |  |  |
|  |  |  |  |  |  |  |  |
|  |  |  |  |  |  |  |  |
|  |  |  |  |  |  |  |  |
|  |  |  |  |  |  |  |  |
|  |  |  |  |  |  |  |  |
|  |  |  |  |  |  |  |  |
|  |  |  |  |  |  |  |  |
|  |  |  |  |  |  |  |  |
|  |  |  |  |  |  |  |  |
|  |  |  |  |  |  |  |  |
|  |  |  |  |  |  |  |  |
|  |  |  |  |  |  |  |  |
|  |  |  |  |  |  |  |  |
|  |  |  |  |  |  |  |  |
|  |  |  |  |  |  |  |  |

Primers used in this study as discussed in text. Restriction sites and mutated bases are shown in lowercase.

| P1 | CACCCTGCAGCGGGCGGAGAATGTC |
| --- | --- |
| P2 | CACTCGCTCTCCAGGAATTGG |
| P3 | TACTTCCAATCCAATTTAGCGTCTTCCGTTTGTCGTC |
| P4 | TCCTCCACTTCCAATTTTAGCGTCCACTGATGATTCAATTATTGGC |
| P5 | TACTTCCAATCCAATTTAGCCATCACAAAGACGCCTC |
| P6 | TCCTCCACTTCCAATTTTAGCGAAGGCACCGTTGCCGGC |
| P7 | TACTTCCAATCCAATTTAGCTGTTGTCGCGCTTTCC |
| P8 | TCCTCCACTTCCAATTTTAGCCTCCGACAGCCGGACCAAG |
| P9 | GATCcatatgCGGTCGTCTCAGTGCTGC |
| P10 | GATCcatatgCTTTTCGCCTGGCCCTGC |
| P11 | GATCagatctATGATCCCCCGGACCTGTGAC |
| P12 | GTACcctaggGTCAGGATACCAACGCCC |
| P13 | GATCcatatgGTCCCATGGTTTCACTTTGG |
| P14 | GATCcatatgCAAGAGCCTCGCTACGAC |
| P15 | GATCagatctATGGGGAACCAATGCTGC |
| P16 | GACTcctaggGCCTACATTCCTGTGTCC |
| P17 | CGGTACCGAGCTCGACTTTCAC |
| P18 | CGTCAGTATTTGGGATGCTG |
| P19 | CCTCACATCACAGATTTGCG |
| P20 | GCCCatcgatGAGAGTGCTTAATACGAACGC |
| P21 | CCCCagatctTCTGAAGACTCAAAGGAAAAG |
| P22 | GTCAgcggccgccGATTACAAAGACGACGACGATAAATGATTAATTAAGACTACGACGAAAGTGATG |
| P23 | CACAGAGCCACAGAAAGTGG |
| P24 | GATCgcggccgcGTCCACTGATGATTCAATTATTGGC |
| P25 | GTACTCAAAATGGATCACCACTcCCCATGGATCTACAATTGCG |
| P26 | CGCAATTGTAGATCCATGGGgAGTGGTGATCCATTTTGAGTAC |
| P27 | GATCagatctATGTCACCTGGCGACCTTC |
| P28 | GATCagatctATGAGGAAAGACGCATCGC |
| P29 | GATCgcggccgcAGGCCGTGACAACGC |
| P30 | GATCgcggccgcCTCCGACAGCCGGACCAAG |
| P31 | GATCagatctatgGCAGGCCGTGACAACGC |
| P32 | GATCggtaccGAGAGTGCTTAATACGAACGC |
| P33 | GATCgcggccgcGATGAAGTGAACACCGTCTC |
| P34 | GATCgcggccgcGAATGCGTCTGTGAAATTTCTC |
| P35 | GATCgcggccgcTTCTTGCATGGGCGTGCG |
